# Pharmacological inhibition of Notch signaling regresses pre-established abdominal aortic aneurysm

**DOI:** 10.1038/s41598-019-49682-0

**Published:** 2019-09-17

**Authors:** Neekun Sharma, Rishabh Dev, Juan de Dios Ruiz-Rosado, Santiago Partida-Sanchez, Mireia Guerau-de-Arellano, Pramod Dhakal, Helena Kuivaniemi, Chetan P. Hans

**Affiliations:** 1 Department of Cardiovascular Medicine, University of Missouri, Columbia, USA; 2 Dalton Cardiovascular Research Center, University of Missouri, Columbia, USA; 3 Medical Pharmacology and Physiology, University of Missouri, Columbia, USA; 4 Center for Microbial Pathogenesis, The Research Institute at Nationwide Children’s Hospital, Columbus, OH USA; 5 School of Health and Rehabilitation Sciences, Medical Laboratory Science Division, The Ohio State University, Columbus, OH USA; 6 Animal Science Research Center, University of Missouri, Columbia, USA; 7 Division of Molecular Biology and Human Genetics, Department of Biomedical Sciences, Stellenbosch University, Cape Town, South Africa

**Keywords:** Aneurysm, Arterial stiffening

## Abstract

Abdominal aortic aneurysm (AAA) is characterized by transmural infiltration of myeloid cells at the vascular injury site. Previously, we reported preventive effects of Notch deficiency on the development of AAA by reduction of infiltrating myeloid cells. In this study, we examined if Notch inhibition attenuates the progression of pre-established AAA and potential implications. Pharmacological Notch inhibitor (N-[N-(3,5-difluorophenacetyl)-L-alanyl]-(S)-phenylglycine t-butyl ester; DAPT) was administered subcutaneously three times a week starting at day 28 of angiotensin II (AngII) infusion. Progressive increase in pulse wave velocity (PWV), maximal intra-luminal diameter (MILD) and maximal external aortic diameter (MEAD) were observed at day 56 of the AngII. DAPT prevented such increase in MILD, PWV and MEAD (P < 0.01). Histologically, the aortae of DAPT-treated *Apoe*^−/−^ mice had significant reduction in inflammatory response and elastin fragmentation. Naked collagen microfibrils and weaker banded structure observed in the aortae of *Apoe*^−/−^ mice in response to AngII, were substantially diminished by DAPT. A significant decrease in the proteolytic activity in the aneurysmal tissues and vascular smooth muscle cells (vSMCs) was observed with DAPT (P < 0.01). In human and mouse AAA tissues, increased immunoreactivity of activated Notch signaling correlated strongly with CD38 expression (R^2^ = 0.61). Collectively, we propose inhibition of Notch signaling as a potential therapeutic target for AAA progression.

## Introduction

Abdominal aortic aneurysm (AAA) is a localized dilation of the abdominal aorta exceeding the normal diameter (~20 mm) by 1.5 times (≥30 mm)^1^. In the United States, AAA accounts for more than 9,000 deaths per year^2^. Pharmacological approaches to limit progression of small AAA (30–50 mm) using anti-inflammatory drugs have not been successful^3,4^. Surgical interventions for the large AAAs (>50 mm) are associated with a significant financial burden and do not provide long-term survival advantages for the small AAAs (≥30–50 mm)^5–7^. These limited options for treatment of AAA highlight the need for innovative research to halt the progression of the disease.

Clinical and experimental research has identified infiltration of immune cells contributing to aneurysm initiation^8,9^. Progression of AAA is accompanied by differentiation of vSMC into a synthetic phenotype in the medial layer, their apoptotic cell death and perpetual expansion of adventitial layer^10,11^. These pathways have emphasized the contribution of aortic vSMCs to aortic wall stiffness via phenotypic changes and extracellular matrix (ECM) dysregulation^12,13^. Previously, we showed that Notch inhibition prevents the development of early AAA in AngII-mouse model by macrophage-dependent mechanisms^14^. Studies by us and others have also established direct effects of Notch1 signaling on vSMCs in vascular diseases^15–17^. Notch signaling has been implicated in vSMCs phenotypic malformations both as a positive and a negative regulator^18,19^. However, the effects of inhibition of Notch signaling on pre-established AAA as a therapeutic target have never been explored.

Hypothesizing that inhibition of Notch signaling will induce regression/stabilization of pre-established aneurysm in an experimental model of AngII-induced AAA, we inhibited Notch signaling at day 28, with or without prolonged AngII infusion. The study was intended to mimic the clinical setting in which a therapeutic drug may be administered in the absence or presence of underlying secondary causes. We demonstrate that Notch inhibition stabilizes pre-established AAA and increases the factors of stability in an experimental model of AngII-induced AAA via a CD38 signaling dependent mechanism.

## Results

### Notch inhibition reverses aortic stiffness and promotes factors of AAA stability

To examine the therapeutic potential of the Notch inhibitor (N-[N-(3,5-difluorophenacetyl)-L-alanyl]-(S)-phenylglycine t-butyl ester; DAPT) on the progression and stability of pre-established AAA, we used an AngII-induced mouse model of AAA in our studies. Transabdominal ultrasound imaging showed a significant increase in the MILD (1.59 ± 0.34 vs. 0.90 ± 0.03 mm, P < 0.001), PWV (1.50 ± 0.28 vs. 0.93 ± 0.04 m/s, P < 0.001), and a decrease in distensibility (74.2 ± 18.8 vs. 103.7 ± 9.99 1/MPa, P < 0.001) and radial strain (24.9 ± 7.89 vs. 53.1 ± 4.2%, P < 0.001) in the *Apoe*^−/−^ mice in response to AngII than controls at day 28 (Supplementary Fig. 2 and data not shown).

**Figure 1:**
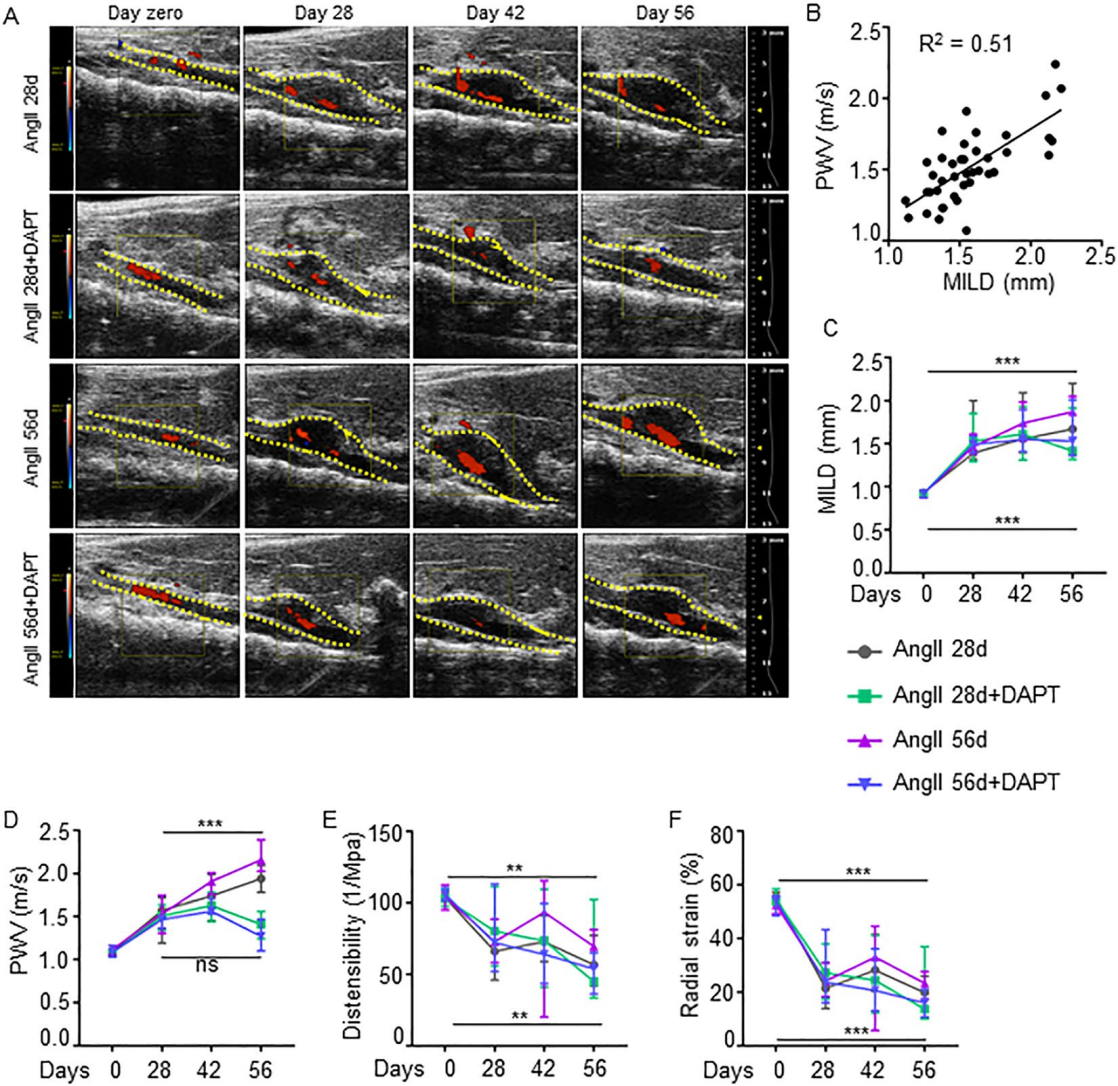
Stabilization of AAA progression by Notch inhibition. (**A**) Representative transabdominal ultrasound images showing the maximal intraluminal diameter (MILD) at day 0, 28, 42 and 56 of indicated experimental groups. DAPT was started at day 28. Dotted yellow lines outline the lumen. (**B**) Graph showing Pearson’s correlation between PWV and MILD from mice receiving AngII and DAPT for 28 days. (**C**) Quantification of MILD in indicated groups as measured by ultrasound (n = 16–18). (**D**–**F**) PWV, distensibility and radial strain at various days of AngII and DAPT treatments as measured by Vevo Vasc analysis (n = 6–8). Student’s t test followed by Bonferroni post hoc analysis was used for the individual time points in (**C**–**F**) and ANOVA followed by Tukey’s multiple comparison analysis . *P < 0.05; **P < 0.01; ***P < 0.001; ns = non-significant.

With the cessation of AngII, no further increase in MILD was observed at day 56 (1.67 ± 0.40 mm; Fig. 1A,C). However, PWV increased progressively and was significantly higher at day 56 (1.94 ± 0.19 m/s) compared to day 28 (Fig. 1D). At day 56, MILD in the DAPT-treated mice (1.41 ± 0.39 mm) was not significantly different compared to AngII 28d. Interestingly, DAPT prevented further increase in PWV (1.42 ± 0.22 m/s) such that it was significantly lower than AngII 28d (P < 0.01; Fig. 1D and Supplementary Fig. 2). Prolonged AngII led to increase in MILD (1.87 ± 0.37 mm) and PWV (2.17 ± 0.20 m/s) at day 56 (P < 0.01). Infusion of DAPT had modest-to-striking effects on MILD and PWV in the aorta with prolonged AngII treatment. Although a marginal increase in MILD (1.53 ± 0.45 mm; Fig. 1C) was observed at day 56, no further progression of PWV (1.27 ± 0.27 m/s, Fig. 1D) was observed and it was significantly lower than AngII 56d (P < 0.01). It is important to distinguish that at day 28, PWV correlated strongly with MILD (R^2^ = 0.51, Fig. 1B), whereas at day 56, the correlation between PWV and MILD was relatively weak (R^2^ = 0.22; Supplementary Fig. 2C). DAPT treatment did not affect distensibility or radial strain significantly (Fig. 1E,F).

**Figure 2:**
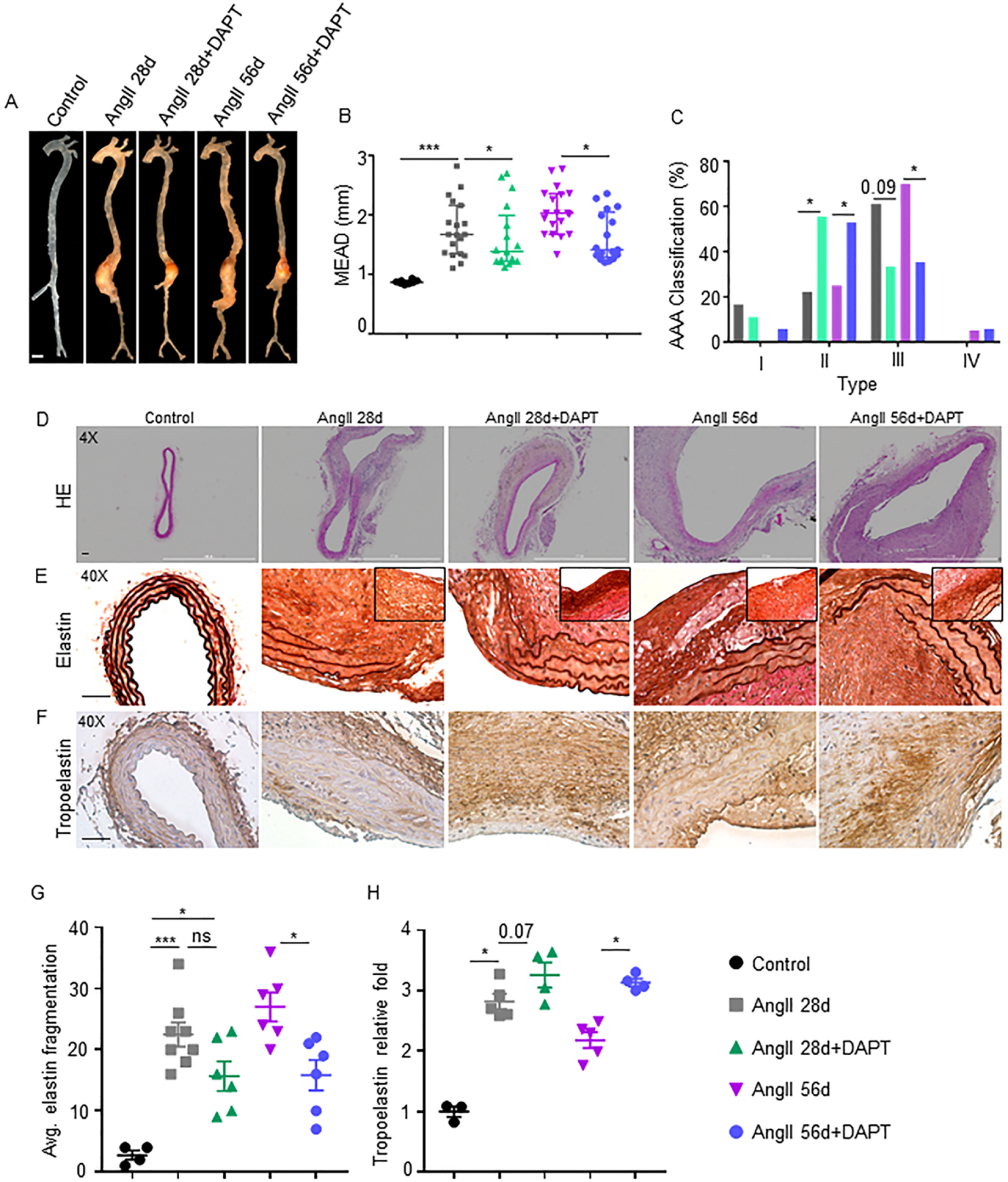
Notch inhibition restores the structural integrity of aorta. (**A**) Representative images showing changes in maximal external aortic diameter (MEAD) of suprarenal aorta in the various experimental groups. (**B**) Quantification of MEAD of suprarenal aorta (mm) as measured by microscopy (n = 16–18). (**C**) Aneurysm severity (type I to IV) scored using a classification system and analyzed by Fisher exact test. (**D**) Representative histological images showing H&E staining in the experimental groups at 56 days of DAPT treatment. (**E**,**F**) Representative immunohistochemistry (IHC) images of aorta showing elastin and tropoelastin staining in the indicated groups. (**G**,**H**) Quantification of elastin fragmentation scores and tropoelastin expression (n = 6). Tukey multiple comparisons test was used for data analysis in (**G-H**). *P < 0.05; ***P < 0.001; ns = non-significant. Scale bar = 1mm in A and 50 µm in (**D–F**).

Consistent with the ultrasound data, the macroscopic examination of aortae at day 56 demonstrated a significantly increased MEAD in response to AngII in both the groups (AngII 28d and AngII 56d) compared to controls (1.67 ± 0.47 and 2.03 ± 0.38 vs. 0.87 ± 0.07 mm respectively; P < 0.05; Fig. 2A,B). The increase in the MEAD in DAPT-treated mice was significantly reduced (1.38 ± 0.55 and 1.41 ± 0.41 mm in AngII 28d + DAPT and AngII 56d + DAPT respectively) as compared to their counterparts (P < 0.05). It is important to note that at day 56, the correlation between PWV and MEAD was not significant (R^2^ = 0.09; Supplementary Fig. 2D). The correlation between MILD and MEAD, however, remained strong in these experimental groups (R^2^ = 0.88; Supplementary Fig. 2E).

At day 56, the AAAs in AngII 28d and AngII 56d were classified as type III in ~60% (11/18) and type II in ~25% (4/18) of the mice (P < 0.05; Fig. 2C). With DAPT treatment, AAAs were classified as ~35% (6/18) as type III and ~55% (10/18) as type II (P < 0.01). Overall mortality and weights were not significantly different among these groups from day 28 to 56 of AngII (data not shown). One mouse in each of the prolonged AngII group died from acute aortic rupture after 28 days as ascertained through post-mortem examination (Fig. 2C).

At day 28 of the AngII, there was marked fragmentation of elastin and loss of vSMCs within the medial layer and a thickened adventitia consisting of inflammatory infiltrate (Supplementary Fig. 3). AAA continued to expand and exhibited complex pathology in AngII 28d and AngII 56d (Fig. 2D–F). Significant disruption of the elastic lamellar architecture in the medial layer was observed at day 56 in AngII 28d (Fig. 2D,E). Prolonged AngII further increased elastin fragmentation in AngII 56d mice (P < 0.001; Fig. 2D,G). DAPT significantly reduced elastin fragmentation compared to their counterparts (P < 0.05; Fig. 2G). More importantly, newly synthesized elastin was increased in the DAPT-treated groups in the vicinity of elastin breaks (inserts in Fig. 2E). Consistent with our previous reports^14^, robust increase in tropoelastin immunostaining was observed in the abdominal aorta of mice treated with DAPT (Fig. 2F,H; P < 0.01). Minimal characteristic features of AAA including aortic remodeling were observed with DAPT treatment in these mice. Elastin crosslinking genes were differentially affected by AngII (Supplementary Fig. 4). The expression of tropoelastin (*Eln1*) was significantly decreased in response to AngII, whereas the expression of *emilin1* and lysyl oxidase (*Lox*) increased significantly. The expression of latent transforming growth factor beta binding protein 1 (*Ltbp1*) remained unaffected with AngII. Notch inhibition increased the gene expression of *Eln1* and *Ltbp1*, but the expression of *Lox* increased marginally. It is interesting that with prolonged infusion of AngII, these protective effects of Notch inhibition on elastin crosslinking genes were diminished (Supplementary Fig. 4). No significant differences in the serum lipid levels were detected with DAPT treatment in these experimental mice at day 56 (Supplementary Fig. 5). Expression of Notch1, its downstream target HeyL and upstream ligand Jagged1 were significantly increased in the AngII treated groups (Supplementary Fig. 6). In accordance within our previous studies, DAPT reduced *Notch1* and *HeyL* expression by more than 50% whereas expression of Jagged1 was marginally reduced^14^. As reported earlier^14^, gastrointestinal toxicity, including goblet cell metaplasia, and dilatation of intestinal crypts/glands was observed in the treated mice by Periodic Acid–Schiff (PAS) staining (data not shown). Interestingly, marginal proliferation of goblet cells and mucosal epithelial necrosis was also observed in the *Apoe*^−/−^ mice treated with AngII (data not shown). Overall, these findings suggested that Notch inhibition at late stage of the disease ameliorate the progressive growth of AAA and also lessened the aortic stiffness of the aorta.

### Notch Inhibition minimizes collagen and ECM degradation

**Figure 3:**
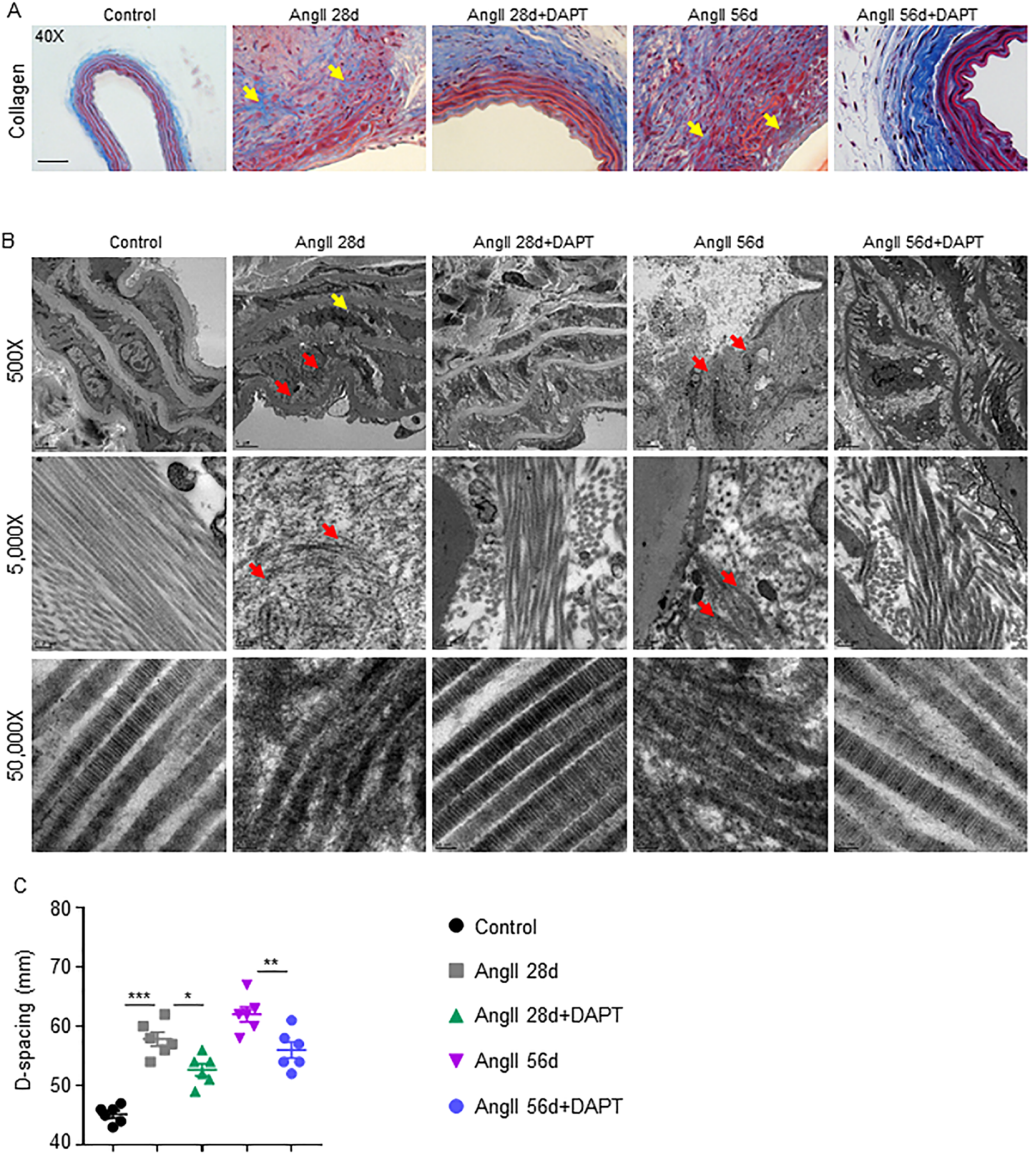
Notch Inhibition minimizes collagen and ECM degradation. (**A**) Representative trichrome images of aorta showing collagen contents in the indicated groups. (**B**) Representative transmission electron microscopy (TEM) images comparing collagen bundles/fibers and D-spacing of collagen molecules. (**C**) Quantification of D-spacing in experimental groups as determined by TEM (n = 4). Tukey multiple comparisons test was used for data analysis in **C**. *P < 0.05; **P < 0.01; ***P < 0.001. Scale bar = 50 µm in **A** and 5 µm in top, 0.2 µm in the middle and 50 nm in the lower panel of B.

Aortic stiffness in AAA is primarily determined by the loss of collagen and ECM-related changes in aortic wall architecture^20^. Medial collagen deposition was observed in the elastin breaks-adjacent segments with AngII infusion and DAPT treatment minimized such deposition (yellow arrows; Fig. 3A).

We previously reported defects in the organization and ultra-architecture of collagen within AAA, which correlate with aortic stiffness^21^. At day 56, abundant naked and abnormal collagen microfibrils with weakened banded structure and undulating longitudinal profile were observed in AngII 28d mice in the region close to elastin disruption (red arrows; middle panel, Fig. 3B). Abnormal endothelial disruption, vacuolization and thickening were also observed in these groups (upper panel; Fig. 3B). Prolonged treatment with AngII (56d) exhibited severe collagen damage and substantial aortic disruption. Interestingly, DAPT reduced the amount of abnormal collagen microfibrils and preserved the elastic lamina along with large areas of dense compacted collagen and differing degrees of preserved banded structure. The aortic tissues from these DAPT treated mice exhibited less endothelial disruption and vacuolization than their counterparts (Fig. 3B). No visible abnormal collagen fibrils were observed in the control mice without AngII.

D-periodic spacing of type I collagen is the key metric of collagen fibril morphology^22^. Increase in d-spacing was observed at day 56 in AngII 28d and AngII 56d mice (57.8 ± 2.86 and 62.0 ± 3.03 nm respectively; Fig. 3C) compared to controls (45.2 ± 1.47 nm; P < 0.01). Notch inhibition significantly lowered the d-spacing (52.6 ± 2.50 and 55.5 ± 3.29 nm), compared to respective counterparts (P < 0.01). Together, these data suggest that the treatment with DAPT results in partial restoration of elastin and collagen fibers, thereby resulting in aneurysm stability.

### Notch inhibition reduces proteolytic activity of aorta and vSMCs

**Figure 4:**
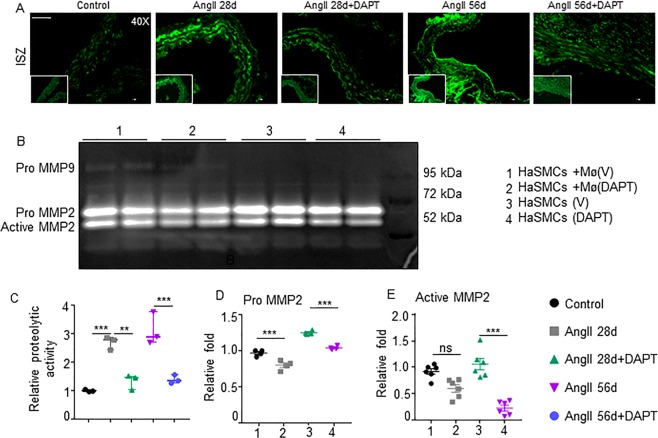
Notch Inhibition decreases proteolytic activity of the aorta and vSMCs. (**A**) Representative *in situ* zymography (ISZ) images showing the proteolytic activity in the aorta. (**B**) MMP2 and MMP9 activity in the HaSMCs cultured alone or with macrophages in the presence and absence of DAPT as determined by gelatin zymography. (**C**) Quantification of proteolytic activity by ISZ (n = 4). (**D**,**E**) Quantification of pro- and active MMP2 from three independent experiments using ImageJ software. Tukey multiple comparisons test was used for data analysis in (**C**,**D**,**F**). Paired two-tailed Student’s t test was used in C-E. **P < 0.01; ***P < 0.001; ns = non-significant. Scale bar = 50 µm in **A**.

Proteolytic activity and loss of vSMCs contribute to the progression of AAA by weakening and stiffening of the medial layer^23^. A significant increase in the matrix metalloproteinase (Mmp) activity in the abdominal aortae was observed in AngII 28d and AngII 56d mice (P < 0.001, Fig. 4A,C) in the regions of elastin breaks. Conversely, DAPT significantly decreased the proteolytic activity, suggesting protective effects against aneurysm propagation in the aortic wall of *Apoe*^−/−^ mice (Fig. 4A,C). The crosstalk between vascular and inflammatory cells contributes to the progression of AAA. To investigate the interaction between vSMCs and macrophages with regard to proteolytic activity, we employed the transwell coculture system. MMP2 activity in these HaSMCs was significantly decreased by direct inhibition of Notch or through Notch-deficient macrophages as determined by gelatin zymography (Fig. 4B,D,E). Interestingly, MMP9 activity was also observed in the HaSMCs cocultured with macrophages and DAPT abolished it (Fig. 4B and Supplementary Fig. 7). Overall, Notch inhibition in the macrophages seems to be sufficient to influence the proteolytic activity of vSMCs through some unknown factors.

### Notch inhibition reduces inflammatory cytokines and synthetic phenotype of vSMCs

**Figure 5:**
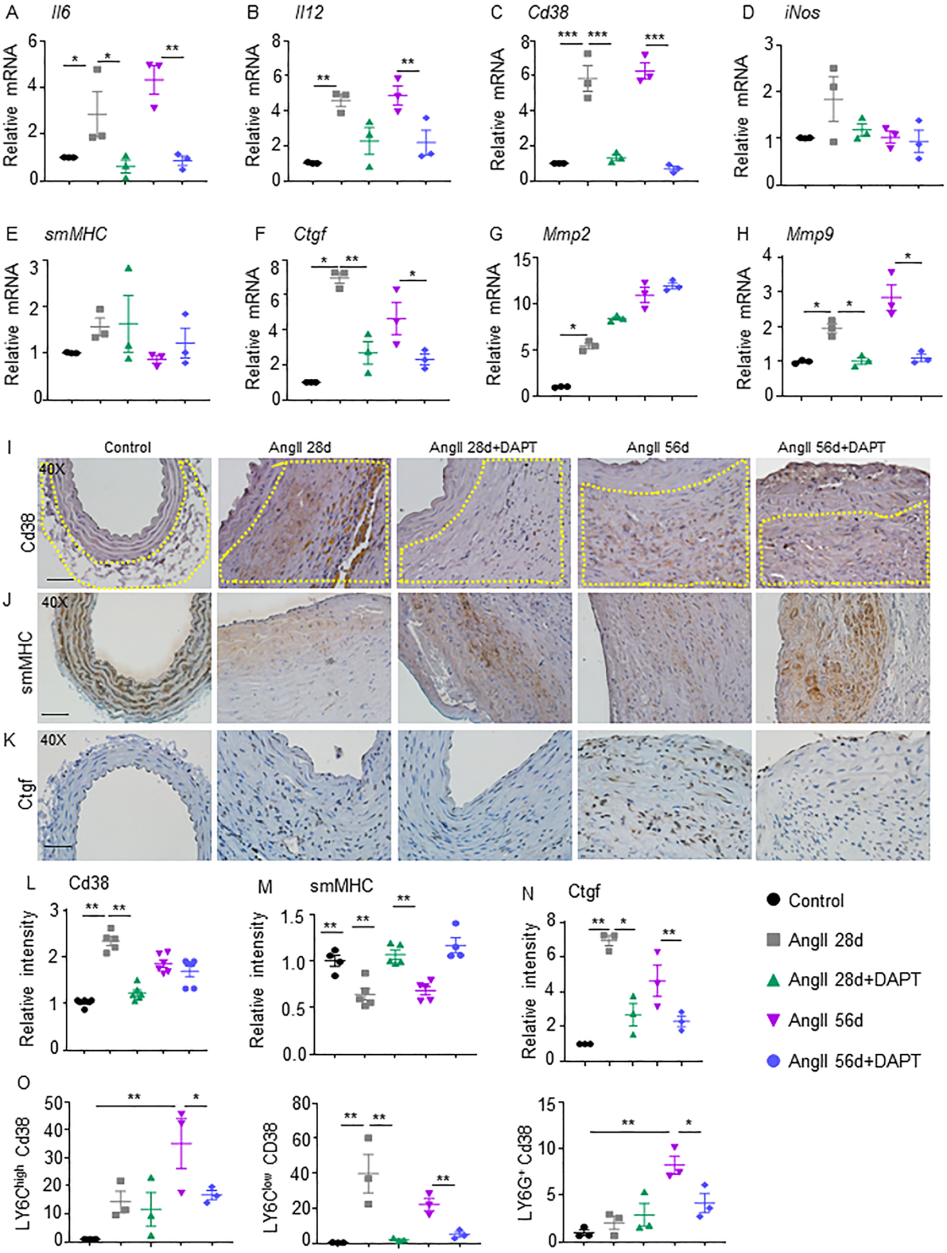
Notch inhibition reduces inflammatory cytokines and synthetic phenotype of vSMCs in the aorta. (**A**–**D**) mRNA expression of inflammatory cytokines (*Cd38*, *Il6*, *Il12* and *iNos*) in the aorta of experimental mice (n = 4). (**E**–**H**) mRNA expression of genes related to vSMC phenotype (*smMHC*, *Ctgf*, *Mmp2* and *Mmp9*) in the aorta of experimental mice (n = 4). (**I**–**K**) Representative IHC images showing immunoreactivity of Cd38 (yellow dotted lines in the adventitial region), smMHC and Ctgf in the suprarenal aorta. (**L**–**N**) Quantification of Cd38, smMHC and Ctgf contents (n = 6). (**O**) Flow cytometry of the abdominal aorta for Cd38 in various subpopulations including CD11b^+^Ly6C^+^ macrophages (left panel), CD11b^+^Ly6C^low^ macrophages (middle panel) and CD11b^+^Ly6G^+^ neutrophils at day 56 of the experimental mice (n = 4). ANOVA followed by Tukey’s multiple comparison analysis was performed for (**A**–**H**,**L–O**). *P < 0.05; **P < 0.01; ***P < 0.001; Scale bar = 50 µm.

To identify factors which could influence proteolytic activity, cDNA from abdominal aorta was analyzed with a panel of inflammatory cytokines and markers of vSMC phenotype. Gene expression of *Ctgf*, *Mmp2*, and *Mmp9* significantly increased in the abdominal aorta of AngII 28d and AngII 56d mice at day 56 compared to control (Fig. 5F–H). DAPT significantly reduced the expression of *Ctgf* and *Mmp9* in both experimental groups. Immunostaining for smMHC revealed that DAPT treatment resulted in partial restoration of vSMCs in the medial layer (P < 0.01; Fig. 5J). Immunostaining of Ctgf on the other hand was significantly reduced with DAPT treatment (P < 0.01; Fig. 5K) at day 56.

Gene expression of *Il6*, *Il12*, and *Cd38* increased significantly in AngII 28d and AngII 56d mice at day 56 compared to controls, whereas, expression of *iNos* was not significantly changed (Fig. 5A–D). With Notch inhibition, significant decrease in the expression of *Il6* and *Cd38*, was observed at day 56 as compared to their respective controls. Expression of *Il12* was decreased with Notch inhibition only in AngII 56d + DAPT. Expression of *iNos*, and M2 genes including *cMyc*, *Egr2*, *Mgl2* and *Tgfβ2* was not significantly altered with AngII or DAPT at day 56 (data not shown).

CD38 pathway has been recently recognized as an intermediate towards activation of antigens in AAA^24^ and it plays a functional role in inflammatory diseases and vSMCs apoptosis^25–28^. At day 56, Notch inhibition led to significantly lower abundance of Cd38 positive cells in the adventitial region of aorta (Fig. 5I,L). Flow cytometry of abdominal aorta at day 56 showed a high percentage of Cd38 positive F4/80^+^/Ly6C^high^ and F4/80^+^/Ly6C^low^ macrophages in AngII 28d and AngII 56d mice (Fig. 5O). Inhibition of Notch significantly lowered Cd38^+^ macrophages in the abdominal vascular wall in both the treatment groups, particularly in the Ly6C^low^ cell population and Ly6G^+^ neutrophils (Supplementary Fig. 8). DAPT had no effect on the expression of Cd38^+^ macrophages from bone marrow, spleen or peripheral blood mononuclear cells from these experimental groups (data not shown). Overall, these data indicate that Notch inhibition-induced protective effects on AAA progression may be associated with inhibition of CD38 signaling.

### Activation of Notch and CD38 signaling in human AAA

**Figure 6:**
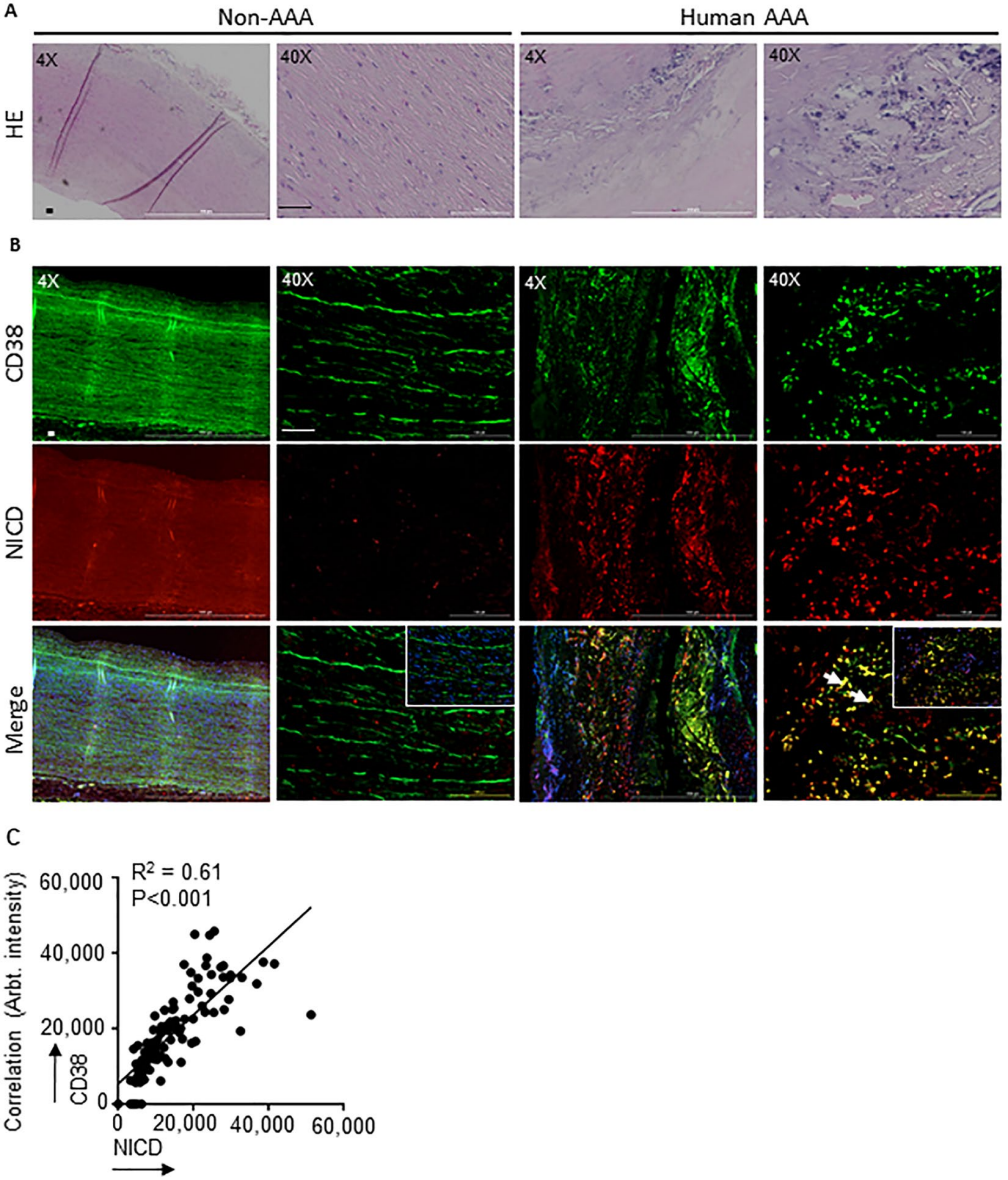
NICD and CD38 expression is upregulated in the aorta of AAA human subjects. (**A**) Representative H&E images of abdominal aortic sections from non-AAA and AAA human subjects (4X and 40X). (**B**) Representative double immunofluorescence images showing NICD and CD38 immunostaining in the aorta of non-AAA and AAA human subjects (4X and 40X). (**C**) Pearson’s correlation graphs showing the coefficient values of NICD/CD38 expression. Scale bar = 50 µm.

Next, we examined the crosstalk of Notch activation with Cd38 signaling in human AAA. Increased immunostaining of NICD was observed in the inflammation-predominant area of AAA compared to abdominal aortic samples from age-matched non-AAA controls (Fig. 6A,B; red). Increased CD38 immunostaining was also observed in these inflammatory regions of tissues from AAA subjects (Fig. 6B; green). Quantification of the co-localization analysis demonstrated a significant increase in the double-positive cells (DPCs) for NICD and CD38 (Fig. 6C, 66.2% in AAA as compared to 4% in controls (R^2^ = 0.61; white arrows in Fig. 6B). Overall, these data demonstrated increased CD38 positive cells in the human AAA, and their linear correlation with Notch signaling.

In summary, our data demonstrate protective roles of Notch inhibition on aortic stiffness and proteolytic activity in a mouse model of AAA at the mid-to-late stage of the disease. Mechanistically, Notch deficiency mediated its protective effects on AAA progression by inactivating the CD38 dependent pathway.

## Discussion

**Figure 7:**
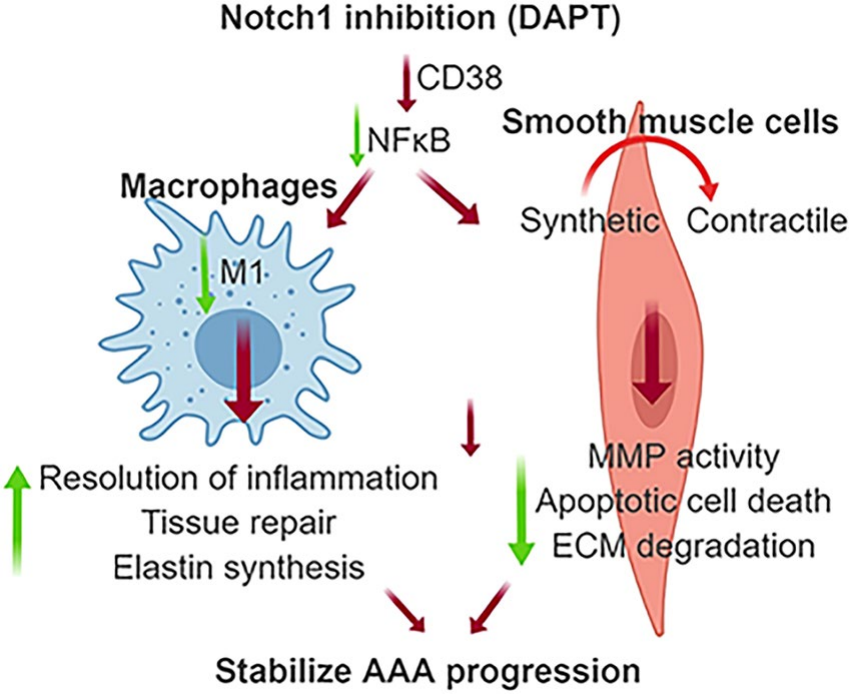
Schematic interpretation of study findings.

The present study was carried out to investigate the therapeutic potential of Notch inhibition on the stability and regression of pre-established AAA using AngII-induced *Apoe*^−/−^ mouse model. DAPT, a potent pharmacological Notch inhibitor was administered at day 28 (late-stage) of AngII infusion and continued for the next 28 days in the absence or presence of prolonged AngII. We provide evidence that Notch inhibition decreases the proteolytic activity and reduces the aortic stiffness without significantly affecting the diameter of the aneurysm. Furthermore, we observed strong correlation of Notch with CD38 signaling, a pro-inflammatory intermediate in human AAA and experimental models. Accordingly, Notch inhibition downregulated CD38 signaling in AAAs and in macrophages. In summary, our studies provide experimental evidence that treatment with Notch inhibitor has the potential to reverse the aortic stiffness and attenuate the progression of the disease and is a potentially viable therapeutic strategy for the AAA, as depicted in our proposed model (Fig. 7).

In addition to the diameter of the AAA, other parameters may provide important information to assess the stability of AAAs. Aortic stiffening is an early change generating aortic wall stress that triggers aneurysmal growth, and remodeling^29^. Measurement of PWV is a reliable and reproducible approach to determine aortic stiffness and seems to be independent of arterial pressure^30,31^. Our novel findings that Notch inhibitor provides stability to the established AAAs by improving the aortic stiffness as primary outcome, have potential therapeutic implications.

AAA progression is attributed to aortic wall degeneration, including elastin fragmentation and immature collagen deposition and proteolytic activity of vSMCs^32^. During the AAA remodeling and ECM repair, the degraded collagen is replaced with stiffer and monomeric collagen fibrils. It is important to note that increased synthesis of ECM proteins including collagens by vSMCs serves an important compensatory role during the early stages of aneurysm development. However, ultrastructural defects in the medial elastic fibers and collagen microfibrils observed in the AngII-treated mice may increase susceptibility to rupture^33^. Accordingly, infusion of DAPT in our studies resulted in partial restoration of elastin and collagen fibers, increased amount of tropoelastin and minimal thickening of the adventitia suggesting a potential mechanism by which Notch inhibition improves aortic stiffness.

We and others have shown that Notch1-dependent phenotypic modulation of vSMCs may also be an important factor to influence vascular pathology^15,34^. Under physiological conditions, vSMCs exist in a contractile state and express a unique repertoire of proteins including α-SMA, SM22α and smMHC that serve as contractile markers^35,36^. Production of profibrogenic cytokines by synthetic vSMCs including CTGF and osteopontin contribute to increased proteolytic activity and defective synthesis of immature ECM components^37^. We and others have demonstrated that preserving or restoring the contractile phenotype of vSMCs attenuates AAA progression^10,15,16^. A strong correlation of aortic stiffness with Mmps is also reported in mouse models of AAA^29^. Proteolytic activity of the vSMC-rich medial layer is greatly modulated at the cellular level by cross-talk among the neighboring cells in the aorta^38^. Macrophages secrete a number of cytokines that mediate complex cell–cell interactions, and maintain and amplify the inflammation cascade in aortic tissue. From the present studies, we infer that infiltration of macrophages into the aorta results in the establishment of a cross-talk among these two types of cells whereby each cell instructs the other to produce increased amounts of MMPs.

CD38, an ADP ribosyl cyclase, is a 45 kDa type II transmembrane protein expressed on the surface of various cells including macrophages and lymphocytes and is involved in the regulation of NF-κB signaling^39,40^. NFκB signaling also regulates the transcription of genes coding for extracellular proteases and vSMC phenotype and could therefore play a role in the aortic remodeling. Based on these observations in human and experimental AAA, we speculate that Notch-mediated CD38 activation might be involved in one of the distinctive and specific aspects of the inflammatory response related to AAA progression. Further studies are required to address the role of CD38 crosstalk with Notch pathway using antibody neutralization or gene deletion strategies in the context of AAA pathogenesis.

Our findings carry a high translational impact as numerous commercially available pharmacological Notch inhibitors including DAPT are already undergoing clinical trials for other conditions. These Notch inhibitors belong to a class of gamma secretase inhibitors (GSIs), and are at different phases of clinical trials in cancer, Alzheimer disease, graft-versus-host diseases and other T cell-mediated human disorders, their roles in AAA are however, largely unknown^41,42^. GSIs are aspartyl proteases responsible for the terminal cleavage of the β-amyloid precursor protein (APP) and more than 90 substrates for γ-secretase inhibitors have been identified^43^. Thus, we cannot rule out the non-targeted effects of DAPT in the context of AAA and future studies are warranted to specifically target Notch1 for mechanistic insights. Blockade of the Notch pathway by DAPT also suppresses neointimal formation and vasculitis in mice^44,45^. Our findings and the recent literature provide new perspectives for targeting the Notch pathway for the potential treatment of effectively limiting AAA progression or the risk of rupture in humans. The presence of apparent adverse effects of DAPT on gastrointestinal toxicity are consistent with our previous studies and literature using the GSIs^14,41^. Overcoming these adverse effects should also be a goal of future research. In conclusion, Notch inhibition reduces the aortic stiffness, which is a measure of AAA progression. Our studies identified a novel mechanism for Notch-induced NFκB activation and interactions between macrophages and vSMCs in the context of AAA. Applications of Notch inhibitors in larger animal models of AAA will give a new perspective for novel target discovery and the development of new strategies to prevent or delay AAA formation.

## Methods

### Mice, aneurysm model, experimental groups and DAPT treatment

All the animal-related experiments were approved by the Animal Care and Use Committee (ACUC #8799) at the University of Missouri (Columbia, MO) and the Institutional Animal Care and Use Committee (IACUC, #AR11-00031) of the Research Institute at Nationwide Children’s Hospital. All the animal experiments conformed to the NIH guidelines (Guide for the Care and Use of Laboratory Animals). Only male mice were studied for the *in vivo* studies because of low incidence of AngII-induced AAA in female mice as described^46^. The ‘arrive guidelines’ were followed to plan the *in vivo* studies. Comprehensive details of all the materials and methods are provided in the Supplemental data. Aneurysmal studies were performed on these mice by infusing AngII following published protocols^47,48^. Mice were anesthetized in a closed chamber with 1–2% isoflurane inhalation in oxygen for 2 to 5 min until immobile. Each mouse was then removed and taped on a heated (37 ± 2 °C) procedure board with 1.0–1.5% isoflurane administered via nosecone during minor surgery. The study was conducted in 4 groups of mice as explained (Supplemental Fig. 1). DAPT (10 mg/kg dissolved in 10% ethanol, 90% corn oil; Sigma-Aldrich) was administered in some of the groups as described^14^. The dose of DAPT for *in vivo* experiments was used in accordance with our previous studies and literature^14,48–50^. At the end of the study, the mice were euthanized by overdose of ketamine (100 mg/kg) and xylazine (20 mg/kg) intraperitoneally according to our previous studies^15^. Blood was drawn and the aortae were perfused with either saline or a fixative depending upon the experiment.

### Transabdominal ultrasound imaging, pulse wave velocity (PWV), distensibility and radial strain measurements

For ultrasonic imaging (ECHO), mice were restrained for <15 seconds to put into the anesthesia chamber, followed by anesthetization with oxygen and vaporized isoflurane (~1–2%). Loss of spinal reflexes were confirmed via toe pinching, and the loss of corneal reflex was assessed by gentle touch of the eye with a soft tissue paper technique. The animals were placed on a heated (41°C) imaging stage in supine position while under anesthesia. The body temperature, heart beat and respiration rates were continuously monitored during the imaging procedure. 40 MHz high-frequency array transducer (Vevo MS550D) was used to collect B-mode, M-Mode, ECG-based kilohertz Visualization (EKV) mode images as well as Power Doppler measurements by the imaging system (Vevo 2100, VisualSonics)^30,48^. *In vivo* aortic stiffness was measured locally in the abdominal aorta by PWV technique by analyzing EKV data collected at various days of AngII infusion using Vevo Vasc software as described previously^30,51^. The measurements for all PWV, distensibility and radial strains were conducted following the two-man principle who were blinded to the study groups.

### AAA classification

AAA complexity was determined by Daugherty’s classification by measurement of the aortic diameter and histological features^52^. Increase in the MEAD by ≥50% was defined as presence of an AAA.

### Serum lipid quantification

Animals were fasted for 4–6 h before blood collection by cardiac puncture as described previously^53^. Serum was separated and lipid analysis was performed by Comparative Clinical Pathology Services in Columbia, Missouri, USA using commercially available assays.

### Histology, immunohistochemistry (IHC) and qRT-PCR

The abdominal aortae from experimental mice were fixed overnight in 10% formalin, rinsed with PBS and processed as described previously^15^. We used low magnification images (4X scale bar) in the HE staining to show the overall aortic remodeling and histologic structures and high magnification (40X) in the IHC to show specific immunostaining for different antibodies. For IHC, the abdominal aortae were stained with antibodies for NICD (1:400 ab8925), CD38 (1:200, AF4947; R & D), smooth muscle myosin heavy chain (smMHC, 1:400, ab53219), connective tissue growth factor (CTGF; 1:400, ab6992), TUNEL (11684795910; Roche) and tropoelastin (1:200; ab21600) as described^15^. The intensity of the immunostaining was evaluated by obtaining 4–5 images from random areas of interest at 40X from each tissue (n = 6–8) and quantified using Fiji version of ImageJ following the software directions^54^. The specificity of all the antibodies was confirmed using appropriate IgG controls in place of primary antibodies at same concentrations as described^30^. Total RNA was extracted from the aortae using the Fibrous RNeasy kit (Qiagen) following the manufacturers’ instructions. Quantitative real-time PCR (qRT-PCR) was performed on CFX connect™ real-time PCR detection system (Biorad) in triplicate. CT for *Rpl13a* was used to normalize gene expression^30^. The primer sequences for genes is detailed in Supplementary Tables 1 and 2. Throughout the study we use gene symbols available from the National Center for Biotechnology Information (NCBI; http://www.ncbi.nlm.nih.gov/).

### Human AAA tissue samples and double immunofluorescence (DIF)

Full-thickness aortic wall tissue specimens were collected from the infrarenal abdominal aorta from patients undergoing AAA repair operations (n = 6; white men aged 60–75 years) at the Harper University Hospital in Detroit, Michigan as described previously^14,55^. The human tissues were obtained after informed consent and approved by the institutional review board of Wayne State University in Detroit, Michigan as described in previous studies^48,55,56^. All the methods in the present study were carried out in accordance with the approved guidelines. Aortic tissue was analyzed for CD38 along with either NFκB or with NICD using DIF. Images were captured using LionHeart fx microscope^57^. Fluorescence intensity was quantified using Gen 5 software (BioTek).

### Coculture assay

Human aortic SMCs (HaSMCs; CC-2571, P5-7, Lonza) were subjected to Notch inhibition through either direct treatment with DAPT or co-cultured with peritoneal macrophages which were pretreated with DAPT for 48 h. HaSMCs were grown in DMEM medium (10569-010; Gibco) containing 10% FBS, 1% penicillin-streptomycin, 4 µg/ml rH Insulin (12585-014; Fisher Scientific), 5 ng/ml recombinant human EGF (PHG0311L; Invitrogen) and 50 µg/ml ascorbic acid (A4544-25G; Sigma)^15^. Peritoneal macrophages were obtained as described^58^. Cell suspension was centrifuged at 4 °C at 2000 rpm for 5 min. Peritoneal macrophages were co-cultured with HaSMCs for 48 h in plain DMEM media. Culture media was processed for gelatin zymography and cells were processed for qRT-PCR analysis.

### Gelatin zymography and *In situ* zymography (ISZ)

Cultured media was concentrated 50-fold using Amicon Ultracel- 10 K centrifugal filter (Millipore, UFC801096) and proteins were separated by electrophoresis, gels were stained with Coomassie blue stain after renaturation/activation and then destained. Images of the zymogram were quantified using ImageJ software^59^. Aortic tissues were cut and incubated with substrate solution containing DQ gelatin (D12054; Invitrogen) and ISZ was performed as described^60^. Negative control sections were treated without DQ gelatin. Sections were mounted with Vectashield medium with DAPI (H-1800; Vector Labs). Fluorescence intensity in the medial layer of the tissue sections was quantified using Gen 5 software (BioTek).

### Transmission electron microscopy (TEM) for mouse aortic tissue samples

Samples were prepared at the Electron Microscopy Core Facility, University of Missouri following a modified version of National Center for Microscopy and Imaging Research (NCMIR) methods for 3D EM^61^. Images were acquired with JEM 1400 transmission electron microscope (JEOL) at 80 kV on Ultrascan 1000 CCD (Gatan, Inc). Difference in length of d-period for collagen fibrils was measured as described previously^62^.

### Single cell preparation for flow cytometric analysis

We isolated the abdominal aortic cells as described previously^15^. Harvested cells were counted per mouse and incubated in saturated doses of anti-mouse Fc receptor in 100 μl of ice-cold FACS buffer (1% bovine serum albumin/0.01% NaN_3_ in PBS) for 15 min. After washing, 2 × 10^6^ cells were stained with various combinations of antibodies in ice-cold FACS buffer for 15 min, and further collected on a LSR II cytofluorometer (BD Biosciences). Gating strategies are shown in Supplementary Fig. 8. Data were analyzed with FlowJo software (Tree Star).

### Statistical analysis

Statistical analyses were performed using GraphPad Prism version 7.0 (GraphPad Software, Inc., CA). Unpaired two-tailed Student’s t test was used to determine statistical difference between two groups for normally distributed continuous variables. For comparison of multiple groups, ANOVA followed by Tukey’s multiple comparison analysis or 2-way ANOVA followed by Bonferroni post hoc tests were used. Data are presented as median ± interquartile range for the PWV, MILD and MEAD. For rest of the quantitation, mean ± SEM was calculated. Pearson’s correlation coefficients were used to calculate correlation analyses. P < 0.05 was considered statistically significant for all tests. The ultrasound and IHC quantitation was done in blinded fashion following the “two-man” principle to ensure scientific precision.

## Supplementary information

Dataset 1
