# Supplementary material for: Pharmacological inhibition of Notch signaling regresses pre-established abdominal aortic aneurysm
Source: Sci Rep. 2019 Sep 17;9:13458. doi: 10.1038/s41598-019-49682-0 (PMC6748927; doi:10.1038/s41598-019-49682-0)

Pharmacological inhibition of Notch signaling regresses pre-established abdominal aortic aneurysm

Neekun Sharma[1,2], Rishabh Dev[1,2], Juan de Dios Ruiz-Rosado[4], Santiago Partida-Sanchez[4], Mireia Guerau-de-Arellano[5], Pramod Dhakal[6], Helena Kuivaniemi[7] , Chetan P. Hans[1,2,3].

[1]Department of Cardiovascular Medicine,

[2]Dalton Cardiovascular Research Center,

[3]Medical Pharmacology and Physiology, University of Missouri, Columbia, USA,

[4]Center for Microbial Pathogenesis, The Research Institute at Nationwide Children's Hospital, Columbus, OH, USA,

[5]School of Health and Rehabilitation Sciences, Medical Laboratory Science Division, The Ohio State University, Columbus, OH, USA,

[6]Animal Science Research Center, University of Missouri, Columbia, USA,

[7]Division of Molecular Biology and Human Genetics, Department of Biomedical Sciences, Stellenbosch University, Cape Town, South Africa.

Supplemental Materials and Methods

Mice, Aneurysm Model, Experimental Groups and DAPT treatment: All the animal-related experiments were approved by the Animal Care and Use Committee at the University of Missouri (Columbia, MO) and the Institutional Animal Care and Use Committee (IACUC) of the Research Institute at Nationwide Children's Hospital. All the animal experiments conformed to the NIH guidelines (Guide for the Care and Use of Laboratory Animals). Only male mice were studied for the *in vivo* studies because of low incidence of AngII-induced AAA in female mice as described[1]. The 'arrive guidelines' were followed to plan the in vivo studies.

Eight-week old, *Apoe*$^{-/-}$ mice (B6.129P2) were purchased from The Jackson Laboratory and inbred to generate sufficient numbers of mice to perform the study in three cohorts (Supplemental figure 1). Mice were kept on a 12 h/12 h light/dark cycle with standard chow. Aneurysmal studies were performed on these mice by infusing AngII following published protocols[2, 3]. Briefly, mice were anesthetized in a closed chamber with 1-2% isoflurane in oxygen for 2 to 5 min until immobile. Each mouse was then removed and taped on a heated (37 ± 2°C) procedure board with 1.0-1.5% isoflurane administered via nosecone during minor surgery. Mini osmotic pumps (Model 2004; Alzet, Cupertino, CA) containing AngII (1000 ng/min/kg; Sigma) were implanted subcutaneously in the neck region of anesthetized mice as described[3].

Because of the linear correlation of progression and stability of aortic aneurysms with aortic stiffness, we focused on pulse wave velocity (PWV; a measure of vascular stiffness) along with maximal intraluminal diameter (MILD) as our primary parameters to determine if Notch inhibition is effective in preventing aneurysmal progression.

To carry out the study, the mice received initial AngII infusion for 28 days and were divided randomly into 4 groups (Supplemental figure 1). Group 1 received vehicle alone (AngII 28d; n=18), Group 2 received DAPT (AngII 28d+DAPT; n=17); Group 3 received additional AngII infusion for 28 days (AngII 56d; n=18) and Group 4 received AngII plus DAPT (AngII 56d+DAPT; n=20). A subset (n=6) of mice was sacrificed at day 28 of AngII infusion.

The mice were monitored daily to assess normal behavior or sudden death. The mice that died in response to AngII were immediately necropsied to determine the cause of death. At the end of the experimental periods, mice were euthanized with an overdose of anesthetics ketamine (100 mg/kg) and xylazine (20 mg/kg). The aortae were dissected, fixed in 10% formalin and cleaned off all the exogenous adipose tissue. Maximal external aortic diameters (MEAD) of the suprarenal aorta were quantified using ZEN lite software (Zen 2.3 blue edition, Zeiss; NY) by an independent researcher *ex vivo* under a microscope as described[5]. One set of tissues was processed for histological and immunohistochemical (IHC) studies (6-9 samples), a second set for transmission electron microscopy (TEM) and *in situ* zymography (ISZ) (6 samples/group), and the third set (5 samples/group) for flow cytometric and quantitative real-time reverse transcriptase PCR (qRT-PCR) analyses as detailed in Supplemental Figure 1. For the MILD and MEAD determination, the data from all mice were included, whereas for the Vevo Vasc analyses, data on 10 mice from 3 subgroups were included.

Transabdominal Ultrasound imaging, PWV, Distensibility and Radial Strain Measurements: For ultrasonic imaging (ECHO), mice were restrained for <15 seconds to put into the anesthesia chamber, followed by anesthetization with oxygen and vaporized isoflurane (~1-2%). Loss of spinal reflexes were confirmed via toe pinching, and the loss of corneal reflex was assessed by gentle touch of the eye with a soft tissue paper technique. The animals were placed on a heated (41°C) imaging stage in supine position while under anesthesia. The body temperature, heart beat and respiration rates were continuously monitored during the imaging procedure. For abdominal aorta measurements, the abdominal hairs were removed by applying

hair removal cream followed by cleaning with wet gauze. Warmed ultrasound gel was applied to the abdominal surface and 40 MHz high-frequency array transducer (Vevo MS550D) was used to collect B-mode, M-Mode, ECG-based kilohertz Visualization (EKV) mode images as well as Power Doppler measurements by the imaging system (Vevo 2100, VisualSonics). *In vivo* aortic stiffness was measured locally in the abdominal aorta by PWV technique by analyzing EKV data collected at various days of AngII infusion using Vevo Vasc software as described previously[6]. Briefly, PWV was determined by simultaneous tracking of R-wave of the ECG and the pulse wave along the two locations of suprarenal abdominal aorta. Vevo Vasc software was used to calculate PWV as a ratio of the distance (d) between two locations along the aorta and time delay (Δt) of the pulse wave between both locations and is expressed in m/s. Similarly, EKV data were analyzed with the Vevo Vasc software to calculate both distensibility and radial strains along the two locations of suprarenal abdominal aorta. The measurements for all PWV, distensibility and radial strains were conducted following the two-man principle who were blinded to the study groups. The corresponding videos of the ECHO for the representative aortae from experimental animals are available upon request.

AAA Classification: AAA complexity was determined by Daugherty's classification by measurement of the aortic diameter and histological features[7]. Type I represents a small single dilation (1.5–2.0 times of a normal diameter); Type II denotes a large single dilation (> 2 times of a normal diameter); Type III is multiple dilations; and Type IV is aortic rupture that leads to death due to bleeding into the peritoneal cavity. Increase in the MEAD by ≥50% was defined as presence of an AAA.

Serum Lipid Quantification: Animals were fasted for 4-6h before blood collection by cardiac puncture as described previously [8]. Serum was separated and lipid analysis was performed by Comparative Clinical Pathology Services in Columbia, Missouri, USA using commercially available assays. All samples were shipped on dry ice and stored frozen until the time of testing.

Histology, IHC and qRT-PCR: The abdominal aortae from experimental mice were fixed overnight in 10% formalin, rinsed with PBS and processed for embedding. Serial sections (5 µM) were prepared by cutting the abdominal aorta into two equal halves and sectioned throughout the tissue. Because of the regional and structural heterogeneity of the aneurysms in AngII-model of AAA, we primarily focused on the regions of elastin breaks to characterize the features of stability in our studies[3]. The sections of the abdominal aorta at regular intervals (200 µM) were subjected to hematoxylin and eosin (H&E), elastin and Masson's trichrome stain for histoarchitectural evaluation of aneurysm as described previously[9]. The serial tissue sections obtained from these mice were further subjected to IHC analyses. For IHC, the abdominal aortae were stained with antibodies for NICD (1;400, abcam), CD38 (1:200, AF4947; R & D), smMHC (1:400, ab53219), connective tissue growth factor (CTGF; 1:400, ab6992), TUNEL (11684795910; Roche) and tropoelastin (1:200; ab21600), as described[9]. The intensity of the immunostaining was evaluated by obtaining 4-5 images from random areas of interest at 40X from each tissue (n=6-8) and quantified using Fiji version of Image J following the software directions[10]. The specificity of all the antibodies were confirmed using appropriate IgG controls in place of primary antibodies at same concentrations as described[5]. To determine the toxicity of Notch inhibitor, liver and intestinal tissue sections were fixed with formalin, dehydrated in graded alcohols, embedded in paraffin wax, sectioned at 5 µm, stained with H&E, and examined by light microscopy. In addition, periodic acid-Schiff (PAS) staining was performed to identify goblet cells as described previously[4]. Total RNA was extracted from aortae using the fibrous RNeasy kit (Qiagen) following the manufacturers' instructions. The concentration of extracted RNA was measured using a Nanodrop ND1000 (Thermo Scientific) to verify that the 260/280 and 260/230 ratios were ~2. qRT-PCR was performed on CFX connect™ real-time PCR detection system (BioRad) in triplicate. A housekeeping gene Rpl13a was used to standardize gene expression

results. The threshold cycle numbers (CT) were computed for each well. For calculating the ΔCT values for each well and processing the results further, the *Rpl13a* amplification CT values were subtracted from the experimental gene CT values for each sample to calculate the ΔCT[14]. The primer sequences for genes used are listed in Supplementary Table 1 and 2. Throughout the study we use gene symbols available from the National Center for Biotechnology Information (NCBI; http://www.ncbi.nlm.nih.gov/)

Human AAA Tissue Samples and Double Immunofluorescence. Full-thickness aortic wall tissue specimens were collected from the infrarenal abdominal aorta from patients undergoing AAA repair operations (n=6; white men aged 60-75 years) at the Harper University Hospital in Detroit, Michigan[4, 11]. Nonaneurysmal infrarenal aortic samples (n=6; white men aged 58-78 years) were collected at autopsies and used as non-AAA controls. Samples were incubated in phosphate-buffered formalin and embedded in paraffin for histological analyses. The human tissues were obtained after informed consent and approved by the institutional review board of Wayne State University in Detroit, Michigan. Aortic tissue was analyzed for CD38 or NICD immunoreactivity using double immunofluorescence (DIF). Images were captured using LionHeart fx microscope (Biotek)[12]. Fluorescence intensity was quantified using Gen 5 software (BioTek). Double positive cells (DPC) with a threshold intensity of 5000 were counted in a colocalization analysis.

Coculture assay: Human aortic SMCs (HaSMCs; cat# CC-2571, P5-7 Lonza) were subjected to Notch inhibition through either direct treatment with DAPT or co-cultured with peritoneal murine macrophages which were pretreated with DAPT for 48 h. HaSMCs were grown in DMEM medium (10569-010; Gibco) containing 10% FBS, 1% penicillin-streptomycin, 4 µg/ml rH Insulin (12585-014; Fisher Scientific), 5 ng/ml recombinant human EGF (PHG0311L; Invitrogen), 50 µg/ml ascorbic acid (A4544-25G; Sigma)[9]. Once the cells became confluent, they were seeded in 12-well plates (30,000 cells/well). Media was changed every other day. Peritoneal macrophages were obtained by injecting thioglycollate (2 ml) in *Apoe*$^{-/-}$ mice and cells were harvested from the peritoneal cavity by flushing with plain RPMI media (10-040-CV; Costar) several times as described[15]. Cell suspension was centrifuged at 4°C at 2000 rpm for 5 min. Cells were then seeded in 12-well plate transwell plate (3462; Costar) in the inserts using RPMI medium containing 10% FBS and 1% penicillin-streptomycin. After 5 h, non-adhered cells were removed by washing. Adhered macrophages were treated with DAPT (10 µM) or DMSO containing media for 48 h. Cells were then serum-starved for 2 h. DMSO or DAPT (10 µM) was added during serum-starvation. Simultaneously HaSMCs cells were also serum-starved for 2 h using plain DMEM media. After completion of incubation time, cells were washed with PBS three times and then peritoneal macrophages containing inserts were put in the plate with HaSMCs at the bottom and further co-cultured for 48 h in plain DMEM media (2 ml/well). After completion of incubation time, culture media was collected and further processed for gelatin zymography.

Gelatin Zymography: Cultured media was concentrated 50-fold using Amicon Ultracel- 10K centrifugal filter (Millipore, UFC801096). Concentrated media samples were mixed with Zymogram sample buffer (BioRad, 161-0764). The proteins were separated in 7.5% polyacrylamide gels containing SDS and 1 mg/ml gelatin (BioRad, 170-6537). After electrophoresis gels were washed (2 x 30 min) with zymogram renaturation buffer (BioRad, 161-0765) while on gentle rotation. Gels were further kept in zymogram development buffer (BioRad, 161-0766) for 30 min at room temperature with gentle shaking. Thereafter gels were kept in development buffer at 37°C with gentle shaking overnight. Next day, gels were stained with Coomassie blue stain and then destained until bands started to appear. Images of the zymogram were captured using gel documentation system (ChemiDoc XRS+; BioRad). Zymograms were quantified using Image J software[16].

***In situ* Zymography (ISZ):** For localization of gelatinolytic activity, ISZ was performed as described by Hadler-Olsen *et al*[17]. Aortic tissues were cryopreserved in OCT medium and stored at -80°C till use. 8 μm thick sections were cut and put on glass slides (12-550-15; Fisher Scientific). Cryosections were air-dried for 15 min and washed in PBS baths (3 x 5 min) to remove traces of OCT medium. Substrate was prepared by dissolving 1 mg DQ gelatin (D12054; Invitrogen) in 1.0 ml of Milli-Q water, and further diluted 1:50 in a reaction buffer containing 50 mM Tris-HCl, 150 mM NaCl, 5 mM $CaCl_2$ and 0.2 mM sodium azide (pH 7.6). 250 μl of this substrate solution was put on top of tissue sections, covered with parafilm and incubated in a humidity chamber at 37°C in the dark for 2 h. Negative control sections were treated in the same way with buffer without DQ gelatin. After completion of incubation, the parafilm was gently removed and the sections were rinsed with Milli-Q water and fixed in 4% buffered formalin-phosphate for 10 min in the dark. Sections were then rinsed in PBS baths (2 x 5 min) and mounted with Vectashield Vibrance antifade medium with DAPI (H-1800; Vector Labs). Images were captured using LionHeart fx microscope (Biotek). Fluorescence intensity in the medial layer of the tissue sections was quantified using Gen 5 software (BioTek).

Transmission Electron Microscopy (TEM) for mouse aortic tissue samples: Samples were prepared following a modified version of National Center for Microscopy and Imaging Research (NCMIR) methods for 3D EM[18]. Unless otherwise stated, all reagents were purchased from Electron Microscopy Sciences and all specimen preparation was performed at the Electron Microscopy Core Facility, University of Missouri. Aortic tissues were fixed in 2% paraformaldehyde, 2% glutaraldehyde in 100 mM sodium cacodylate buffer (pH 7.35). Next, fixed tissues were rinsed with 100 mM sodium cacodylate buffer (pH 7.35) containing 130 mM sucrose. Secondary fixation was performed using equal parts of 4% osmium tetroxide and 3% potassium ferrocyanide in cacodylate buffer and incubated on ice for 1 h, then rinsed with cacodylate buffer and further with distilled water. En block staining was performed for 1 h in a 1% thiocarbohydrazide solution followed by distilled water rinses. Rinsed tissues were incubated in a 2% aqueous osmium tetroxide solution for 30 min at room temperature, and then rinsed with distilled water. Additional en bloc staining was performed using 1% aqueous uranyl acetate and incubated at 4°C overnight, then rinsed with distilled water. A final en bloc staining was performed using Walton's Lead Nitrate solution for 30 min at 60°C. Tissues were rinsed and dehydrated using ethanol, transitioned into acetone, and then infiltrated with Durcapan resin and polymerized at 60°C overnight. Sections were cut to a thickness of 85 nm using an ultramicrotome (Ultracut UCT, Leica Microsystems) and a diamond knife (Diatome). These sections were post-stained using Sato's triple lead solution[19] and 5% aqueous uranyl acetate. Images were acquired with JEM 1400 transmission electron microscope (JEOL) at 80 kV on Ultrascan 1000 CCD (Gatan, Inc). Difference in length of d-period for collagen fibrils was measured as described previously[20, 21].

Single Cell Preparation for Flow Cytometric Analysis: We isolated the abdominal aortic cells as described previously[9]. After the mouse was anesthetized, the abdominal aorta was dissected, minced with fine scissors, and then enzymatically digested with a cocktail of type IV collagenase (0.5 mg/ml) and DNase I (0.1 mg/ml) for 30 min at 37°C with gentle agitation. After digestion, the tissue was passed through 70 μm Nylon Mesh (Fisher Scientific) to make single-cell suspensions. The cells were then lysed with ACK lysing buffer (GIBCO, Life technologies) to lyse red blood cells. Harvested cells were counted per mouse and incubated in saturated doses of anti-mouse Fc receptor in 100 μl of ice-cold FACS buffer (1% bovine serum albumin/0.01% NaN_3 in PBS) for 15 min. After washing, 2 x 10^6 cells were stained with various combinations of antibodies in ice-cold FACS buffer for 15 min, and further collected on a LSR II cytofluorometer (BD Biosciences). Gating strategies for cells are shown in Supplemental figure 7 in the online-only Data Supplement. Blue-fluorescent reactive dye L23105 (Life Technologies) was used to identify dead cells. Absolute cell numbers were calculated with the total cell count

multiplied successively by the percentages for the appropriate gates obtained through flow cytometry.

Intracellular Staining: Single-cell suspensions were washed with complete RPMI 1640 media and adjusted to $3x10^6$ cells/ml. Two ml per well of the cell suspension were incubated in 24-well plate and incubated for 6 h in RPMI 1640 media supplemented with 5% FCS, and 1 µl/ml Golgi Plug (BD Pharmingen) at 37°C, with 5% CO_2. The cells were harvested and washed with FACS buffer and stained with appropriate antibodies. In our protocol, we used Cd38 (clone 90, Biolegend), Tnf-α (MP6-XT22) and iNos (CXNFT; Thermo Scientific) according to the protocol of the Cytofix/Cytoperm kit (BD Biosciences). Data were analyzed with FlowJo software (Tree Star).

Statistical Analysis: Statistical analyses were performed using GraphPad Prism version 7.0 (GraphPad Software, Inc.). All data were assessed for normality and equal variance using GraphPad Prism. Continuous data were examined by Shapiro-Wilk test for normality. Unpaired two-tailed Student's *t* test was used to determine statistical difference between two groups for normally distributed continuous variables. Based on our previous work and reports from other laboratories using this definition, we powered the study with our main outcome variable of MEAD using α=0.05. We estimated our sample size for this variable to be 16 per group to determine if the data are statistically significant assuming a 5% significance level using the t-test. For comparison of multiple groups, ANOVA followed by Tukey's multiple comparison analysis or 2-way ANOVA followed by Bonferroni post hoc tests were used. All data are presented as median ± SEM for the PWV, MILD and MEAD. For rest of the quantifications, mean ± SEM was calculated. Pearson's correlation coefficients were used to calculate correlation between two variables. $P<0.05$ was considered statistically significant for all tests. The ultrasound and IHC quantitation were done in blinded fashion following the "two-man" principle to ensure scientific precision.

References

1. Robinet P, Milewicz DM, Cassis LA, Leeper NJ, Lu HS and Smith JD. Consideration of Sex Differences in Design and Reporting of Experimental Arterial Pathology Studies-Statement From ATVB Council. *Arterioscler Thromb Vasc Biol*. 2018;38:292-303.
2. Daugherty A, Manning MW and Cassis LA. Angiotensin II promotes atherosclerotic lesions and aneurysms in apolipoprotein E-deficient mice. *J Clin Invest*. 2000;105:1605-12.
3. Hans CP, Koenig SN, Huang N, Cheng J, Beceiro S, Guggilam A, Kuivaniemi H, Partida-Sanchez S and Garg V. Inhibition of Notch1 signaling reduces abdominal aortic aneurysm in mice by attenuating macrophage-mediated inflammation. *Arterioscler Thromb Vasc Biol*. 2012;32:3012-23.
4. Cheng J, Koenig SN, Kuivaniemi HS, Garg V and Hans CP. Pharmacological inhibitor of notch signaling stabilizes the progression of small abdominal aortic aneurysm in a mouse model. *J Am Heart Assoc*. 2014;3:e001064.
5. Sharma N, Dev R, Belenchia Anthony M, Aroor Annayya R, Whaley-Connell A, Pulakat L and Hans Chetan P. Deficiency of IL12p40 (Interleukin 12 p40) Promotes Ang II (Angiotensin II)–Induced Abdominal Aortic Aneurysm. *Arteriosclerosis, Thrombosis, and Vascular Biology*. 0:ATVBAHA.118.311969.
6. Sehgel NL, Zhu Y, Sun Z, Trzeciakowski JP, Hong Z, Hunter WC, Vatner DE, Meininger GA and Vatner SF. Increased vascular smooth muscle cell stiffness: a novel mechanism for aortic stiffness in hypertension. *American Journal of Physiology - Heart and Circulatory Physiology*. 2013;305:H1281-H1287.
7. Rateri DL, Howatt DA, Moorleghen JJ, Charnigo R, Cassis LA and Daugherty A. Prolonged infusion of angiotensin II in apoE(-/-) mice promotes macrophage recruitment with continued expansion of abdominal aortic aneurysm. *Am J Pathol*. 2011;179:1542-8.
8. Oumouna-Benachour K, Hans CP, Suzuki Y, Naura A, Datta R, Belmadani S, Fallon K, Woods C and Boulares AH. Poly(ADP-ribose) polymerase inhibition reduces atherosclerotic plaque size and promotes factors of plaque stability in apolipoprotein E-deficient mice: effects on macrophage recruitment, nuclear factor-kappaB nuclear translocation, and foam cell death. *Circulation*. 2007;115:2442-50.
9. Sachdeva J, Mahajan A, Cheng J, Baeten JT, Lilly B, Kuivaniemi H and Hans CP. Smooth muscle cell-specific Notch1 haploinsufficiency restricts the progression of abdominal aortic aneurysm by modulating CTGF expression. *PloS one*. 2017;12:e0178538.
10. Schindelin J, Arganda-Carreras I, Frise E, Kaynig V, Longair M, Pietzsch T, Preibisch S, Rueden C, Saalfeld S, Schmid B, Tinevez JY, White DJ, Hartenstein V, Eliceiri K, Tomancak P and Cardona A. Fiji: an open-source platform for biological-image analysis. *Nat Methods*. 2012;9:676-82.
11. Hinterseher I, Erdman R, Elmore JR, Stahl E, Pahl MC, Derr K, Golden A, Lillvis JH, Cindric MC, Jackson K, Bowen WD, Schworer CM, Chernousov MA, Franklin DP, Gray JL, Garvin RP, Gatalica Z, Carey DJ, Tromp G and Kuivaniemi H. Novel pathways in the pathobiology of human abdominal aortic aneurysms. *Pathobiology : journal of immunopathology, molecular and cellular biology*. 2013;80:1-10.
12. Smith KM, Tsimbalyuk S, Edwards MR, Cross EM, Batra J, Soares da Costa TP, Aragão D, Basler CF and Forwood JK. Structural basis for importin alpha 3 specificity of W proteins in Hendra and Nipah viruses. *Nature Communications*. 2018;9:3703.
13. Jablonski KA, Amici SA, Webb LM, Ruiz-Rosado Jde D, Popovich PG, Partida-Sanchez S and Guerau-de-Arellano M. Novel Markers to Delineate Murine M1 and M2 Macrophages. *PloS one*. 2015;10:e0145342.
14. Yuan JS, Reed A, Chen F and Stewart CN, Jr. Statistical analysis of real-time PCR data. *BMC bioinformatics*. 2006;7:85-85.

15. Oumouna M, Datta R, Oumouna-Benachour K, Suzuki Y, Hans C, Matthews K, Fallon K and Boulares H. Poly(ADP-ribose) polymerase-1 inhibition prevents eosinophil recruitment by modulating Th2 cytokines in a murine model of allergic airway inflammation: a potential specific effect on IL-5. *J Immunol*. 2006;177:6489-96.
16. Schneider CA, Rasband WS and Eliceiri KW. NIH Image to ImageJ: 25 years of image analysis. *Nat Methods*. 2012;9:671-5.
17. Hadler-Olsen E, Fadnes B, Sylte I, Uhlin-Hansen L and Winberg J-O. Regulation of matrix metalloproteinase activity in health and disease. *The FEBS Journal*. 2011;278:28-45.
18. Kantor A, Grant D, Balaraman V, White T and Franz A. Ultrastructural Analysis of Chikungunya Virus Dissemination from the Midgut of the Yellow Fever Mosquito, Aedes aegypti. *Viruses*. 2018;10:571.
19. Sato T. A modified method for lead staining of thin sections. *J Electron Microsc (Tokyo)*. 1968;17:158-9.
20. Tonniges JR, Albert B, Calomeni EP, Roy S, Lee J, Mo X, Cole SE and Agarwal G. Collagen Fibril Ultrastructure in Mice Lacking Discoidin Domain Receptor 1. *Microscopy and microanalysis : the official journal of Microscopy Society of America, Microbeam Analysis Society, Microscopical Society of Canada*. 2016;22:599-611.
21. Tonniges JR, Albert B, Calomeni E, Hans C and Agarwal G. Ultrastructural Imaging of Collagen Fibrils in Mouse Model of Abdominal Aortic Aneurysm. *Microscopy and Microanalysis*. 2016;22:1196-1197.
22. Bhamidipati CM, Mehta GS, Lu G, Moehle CW, Barbery C, DiMusto PD, Laser A, Kron IL, Upchurch GR, Jr. and Ailawadi G. Development of a novel murine model of aortic aneurysms using peri-adventitial elastase. *Surgery*. 2012;152:238-246.

Supplementary Table 1: List of primer sequences used for qRT-PCR studies for Mus musculus

Gene	Forward Primer (5'-3')	Reverse Primer (5'-3')
Il12	FGG AAG CAC GGC AGC AGA ATA	AAC TTG AGG GAG AAG TAG GAA TGG
iNos	CTC GGA GGT TCA CCT CAC TGT	TCC TGA TCC AAG TGC TGC AGA
Cd38	TTG CAA GGG TTC TTG GAA AC	CGC TGC CTC ATC TAC ACT CA
Mmp2	GAT GTC GCC CCT AAA ACA GA	TGG TGT TCT GGT CAA GGT CA
Mmp9	AGA CCT GAA AAC CTC CAA CCT CAC	TGT TAT GAT GGT CCC ACT TGA GGC
SmMHC	GGT CGT GGA GTT GGT GGA AA	CTG CCA TGT CCT CCA CCT TAG
Ctgf	CTT CTG CGA TTT CGG CTC C	TGC TTT GGA AGG ACT CAC CG
Il6	CTA CCC CAA TTT CCA ATG CT	ACC ACA GTG AGG AAT GTC CA
Lox	CAG CCA CAT AGA TCG CAT GGT	GCC GTA TCC AGG TCG GTT C
Emilin1	TGTGCCTACGTGGTGACTC	GGT ACA TGA TAC TTC GGG AAC AG
Ltbp1	GGT CGC ATC AAG GTG GTC TTT	GTG GTG GTA TTC CCC TTC TGG
Eln1	CTT TGG ACT TTC TCC CAT TTA TCC	GGT CCC CAG AAG ATC ACT TTC TC
Notch1	CCG TTA CAT GCA GCA GTT TC	AGC CAG GAT CAG TGG AGT TG
HeyL	GGA ACA ACA GAG AAT GAA CCA ACC	TCT TGA CTT TGC CAG GGA TTA GC
Jagged 1	GGC TTC TCA CTC AGG CAT GAT A	GTG GGC AAT CCC TGT GTT TT
Rpl13a	TCC CTG CTG CTC TCA AGG	GCC CCA GGT AAG CAA ACT T

Supplemental Table 2: List of human primer sequences used for qRT-PCR studies

Gene	Forward Primer (5'-3')	Reverse Primer (5'-3')
MMP2	ACG ACC GCG ACA AGA AGT AT	ATT TGT TGC CCA GGA AAG TG
Notch1	GCA GTT GTG CTC CTG AAG AA	CGG GCG GCC AGA AAC
Col1α1	CAG CCG CTT CAC CTA CAG C	TTT TGT ATT CAA TCA CTG TCT TGC C
CTGF	CTC CAC CCG GGT TAC CAA TG	CCG GGA CAG TTG TAA TGG CA

Supplemental Figure 1. Schematic for the *in vivo* Studies.

28 days post AngII infusion, mice were randomly divided into 4 subgroups. Group 1 received vehicle alone (AngII 28d; n=18), Group 2 received DAPT (AngII 28d+DAPT; n=17); Group 3 received additional AngII infusion for 28 days (AngII 56d; n=18) and Group 4 received AngII plus DAPT (AngII 56d+DAPT; n=20). A subset (n=6) of mice was sacrificed at day 28 of AngII infusion. Control group received only saline/vehicle throughout the study (n=6).

Supplemental Figure 2. Notch inhibition decreases aortic stiffness in mid- and late-stage aneurysm.

(A) MILD and PWV at days 28, 42 and 56 in the indicated experimental groups as measured by ultrasound followed by Vevo Vasc analysis. **(C-E)** Graphs showing Pearson's correlation between PWV and MILD **(C)** PWV and MEAD **(D)** and MEAD and MILD **(E)** from mice receiving AngII and DAPT for 56 days. Student's *t*-test followed by Bonferroni post hoc analysis was used for the individual time points. Tukey multiple comparisons test was used for data analysis. *$P<0.05$; **$P<0.01$; ***$P<0.001$.

Supplemental Figure 3. Loss of vSMCs at day 28 of AngII in the medial layer of aorta.

Representative images of *Apoe*$^{-/-}$ mouse aorta showing adventitial thickening (H&E staining), structural changes (elastin, collagen and smMHC staining) and apoptosis of vSMCs (TUNEL) at day 28 of AngII infusion. Scale bar = 50 μm.

Supplemental Figure 4. Notch inhibition increases elastin biosynthesis-realed genes.

(A-D) mRNA expression of elastin biosynthesis genes (*Eln1, Emilin1, Lox* and *Ltbp1*) in the aorta of experimental mice at day 56 (n=3). *P<0.05; **P<0.01; ***P<0.001; ns = non-significant in paired two-tailed Student's *t*-test.

Supplemental Figure 5. DAPT does not affect serum lipid levels at day 56.

Graphs showing serum cholesterol **(A)**, triglycerides **(B)** and HDL cholesterol **(C)** measured in experimental mice at day 56 in mice with and without AngII (n=4 per group).

Supplemental Figure 6. DAPT decreases NICD expression. (A-C) mRNA expression of *Notch1*, *HeyL* and *Jagged1* in the aorta of experimental mice at day 56 (n=6). **(D)**, Representative IHC images of NICD immunostaining in abdominal aortic sections from indicated experimental groups (40X). *P<0.05; **P<0.01; ***P<0.001 in paired two-tailed Student's *t*-test.

Supplemental Figure 7. Multiple exposures of the gelain zymography from co-culture experiment showing low, medium and high contrast.

Supplemental Figure 8. Gating strategy of macrophages from the abdominal aorta. (**A)** Representative dot plot.

Supplementary Figure 1

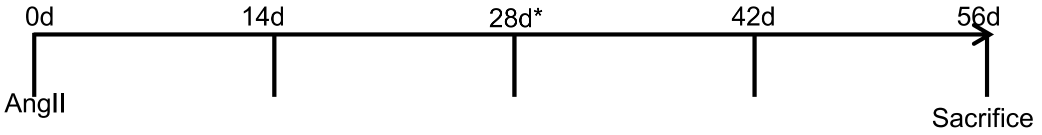

*At day 28 of AngII, divided into 4 groups:
- AngII 28d (n=18)
- AngII 28d+DAPT (n=17)
- AngII 56d (n=18)
- AngII 56d+DAPT (n=20)

*One set was sacrificed at day 28
Control mice received saline/vehicle for 56d (n=6)

The study was performed in three sets:
Set 1: Histology and immunohistochemistry (n =6-9 samples)
Set 2: Transmission electron microscopy and *in situ* zymography (n=6 samples)
Set 3: flow cytometry and quantitative real-time reverse transcriptase PCR (n=5 samples)

Schematic for the in vivo Studies

Supplementary Figure 2

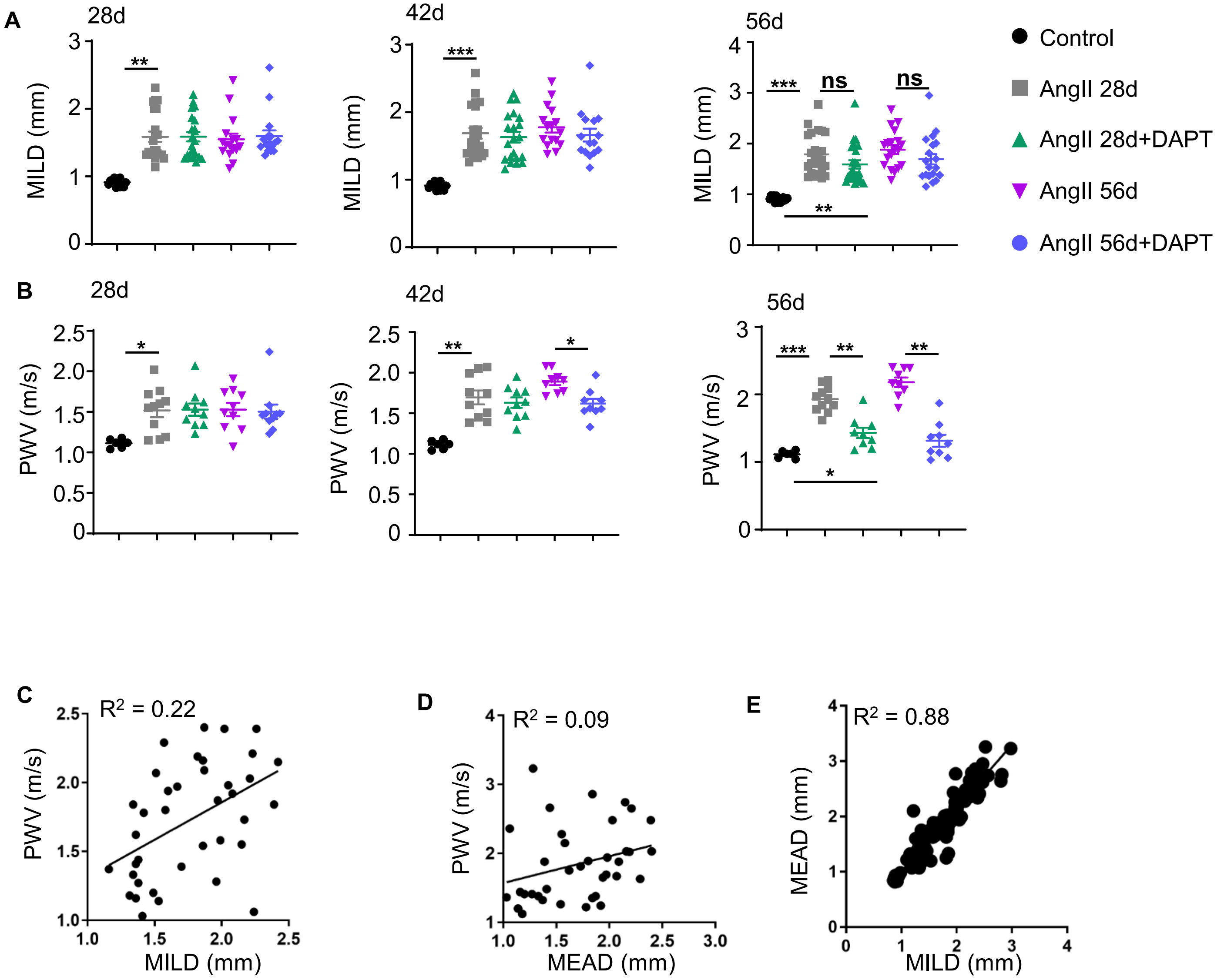

Supplementary Figure 3

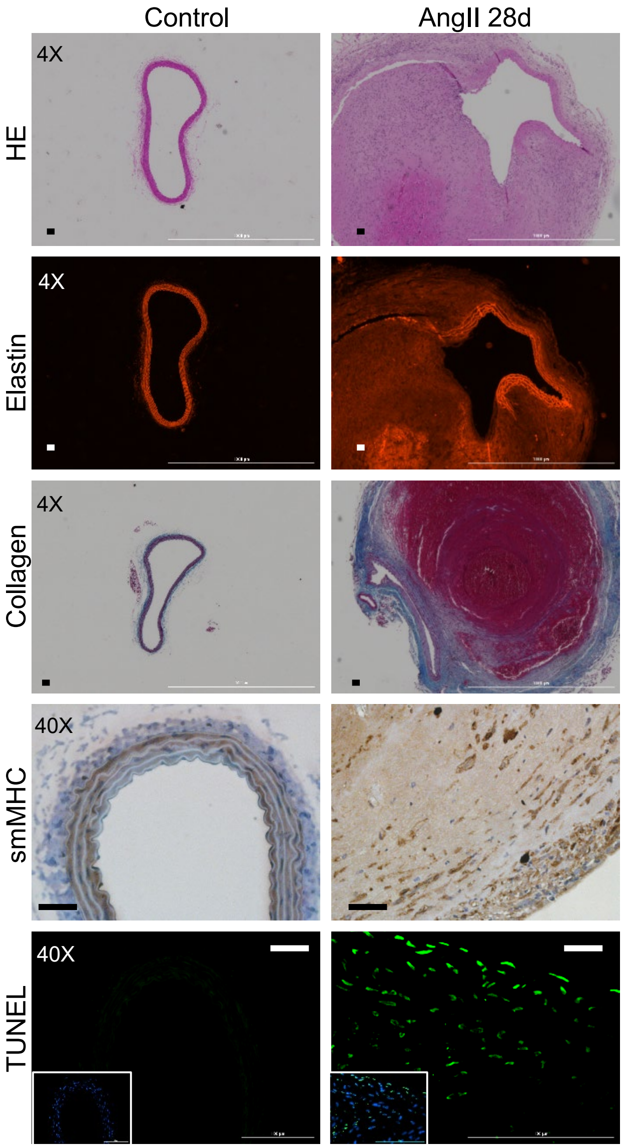

Supplementary Figure 4

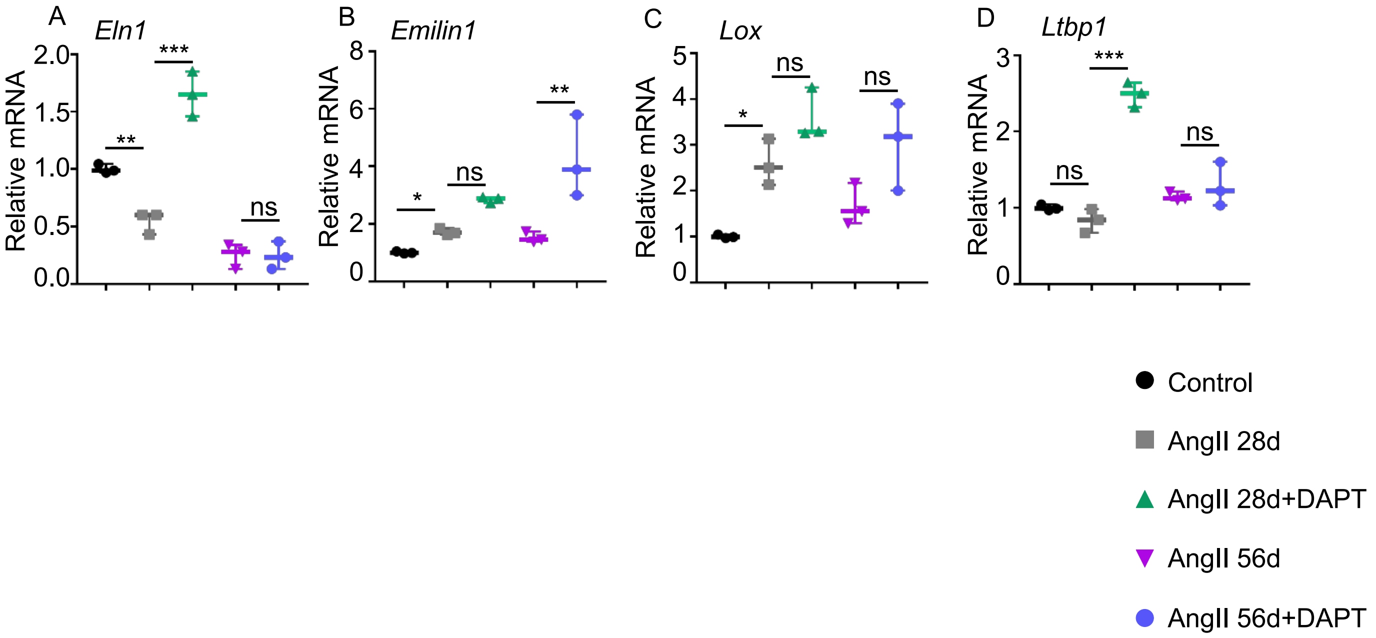

Supplementary Figure 5

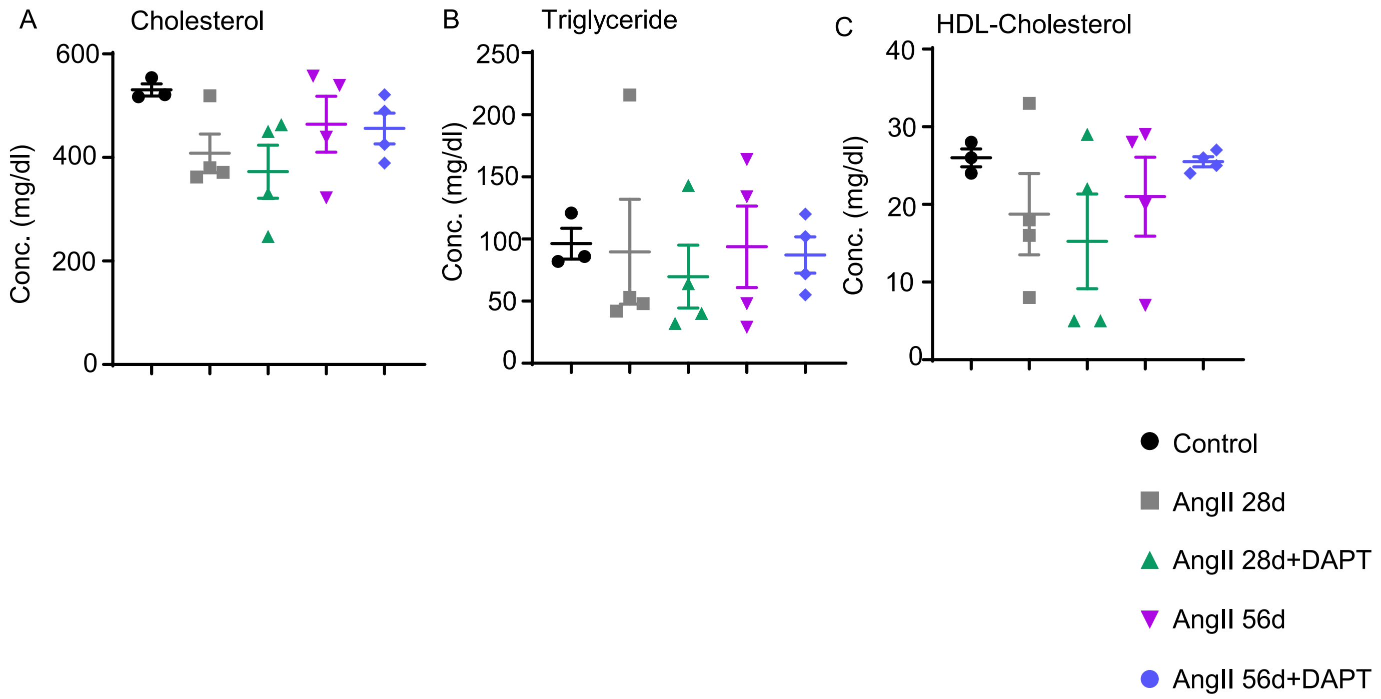

Supplementary Figure 6

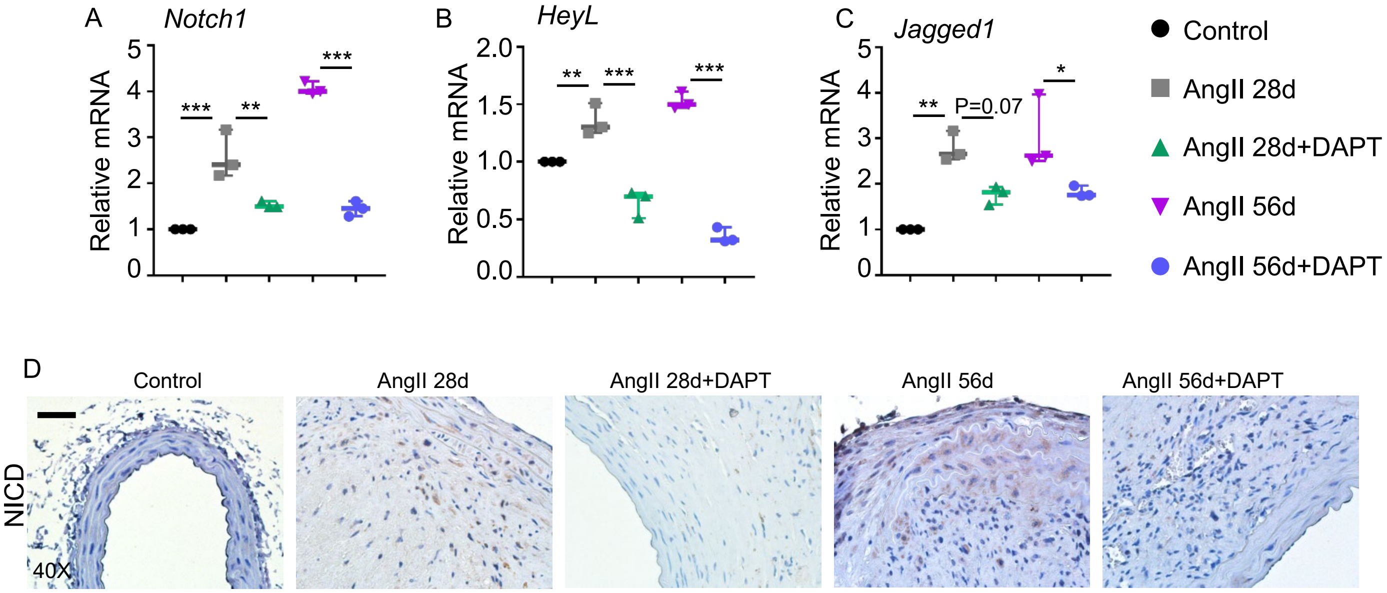

Supplementary Figure 7

1 HaSMCs +Mø(V)
2 HaSMCs +Mø(DAPT)
3 HaSMCs (V)
4 HaSMCs (DAPT)

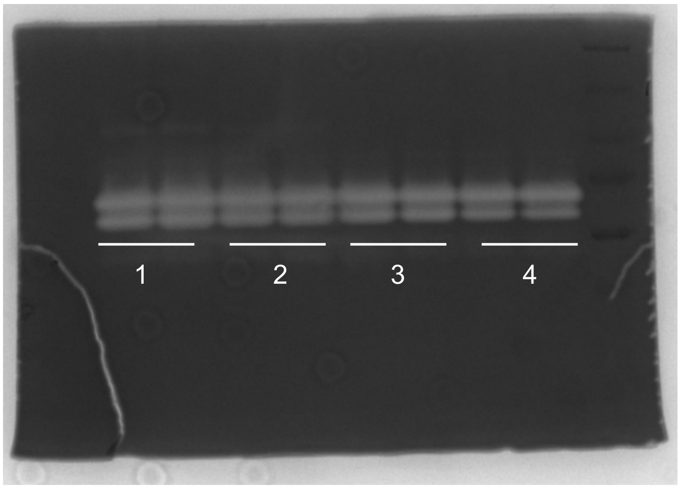

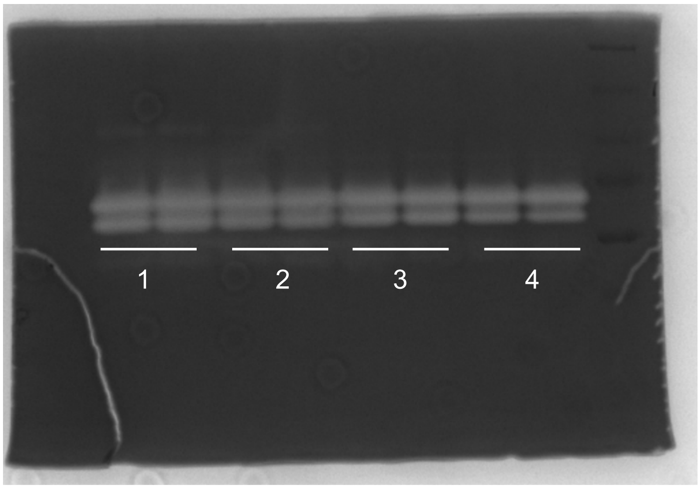

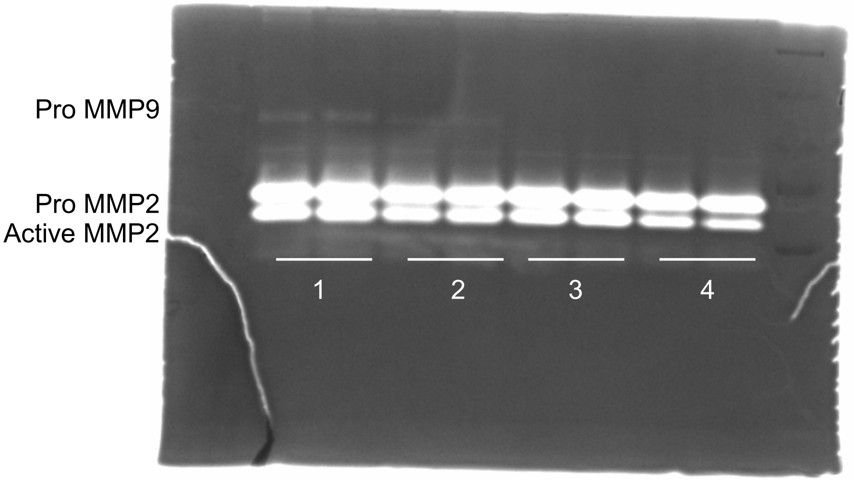

Supplementary Figure 8

A

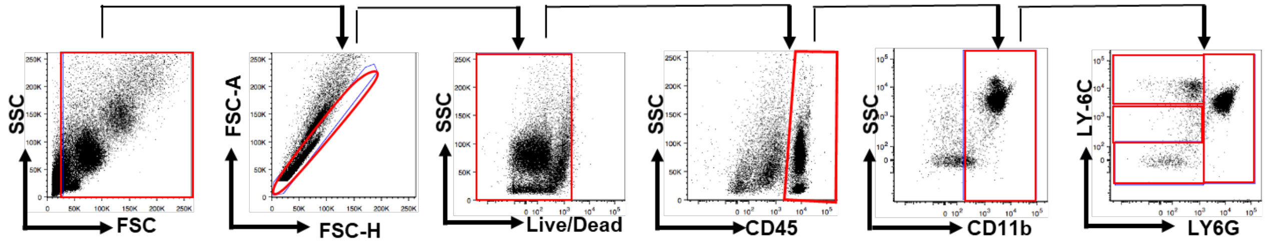

Supplement: Supplementary file 1 — Dataset 1 [file 41598_2019_49682_MOESM1_ESM.pdf]
